# Pitavastatin is a novel Mcl-1 inhibitor that overcomes paclitaxel resistance in triple-negative breast cancer

**DOI:** 10.1186/s40164-025-00716-6

**Published:** 2025-10-22

**Authors:** Dongmi Ko, Soeun Park, Minsu Park, Seongjae Kim, Jung Min Park, Juyeon Seo, Kee Dal Nam, Yong Koo Kang, Lee Farrand, Eunsun Jung, Yoon-Jae Kim, Ji Young Kim, Jae Hong Seo

**Affiliations:** 1 Division of Medical Oncology, Department of Internal Medicine, Korea University College of Medicine, Korea University, Guro Hospital Campus, 148 Gurodong-ro, Guro-gu, Seoul, 02841 Republic of Korea; 2 Brain Korea 21 Program for Biomedical Science, Korea University College of Medicine, Korea University, Guro Hospital Campus, 148 Gurodong-ro, Guro-gu, Seoul, 02841 Republic of Korea; 3 Department of Biomedical Research Center, Korea University Guro Hospital, Korea University, Guro Hospital Campus, 148 Gurodong-ro, Guro-gu, Seoul, 08308 Republic of Korea; 4 Adelaide Medical School, Faculty of Health and Medical Sciences, The University of Adelaide, Adelaide, SA 5000 Australia

**Keywords:** Pitavastatin, Mcl-1, Triple-negative breast cancer, Paclitaxel resistance, Cancer stem cells, Drug repurposing, Metastasis

## Abstract

**Background:**

Triple-negative breast cancer (TNBC) is notorious for its poor prognosis, high metastatic rates, and resistance to chemotherapy. We sought to investigate the anticancer effects of pitavastatin (PITA), a promising candidate for drug repurposing due to its potent inhibition of myeloid cell leukemia 1 (Mcl-1).

**Methods:**

The impact of PITA on TNBC cells was assessed in vitro by examining cell viability, apoptosis, mitochondrial function, and effects on cancer stem cell (CSC) properties. The interaction between PITA and Mcl-1 was explored using molecular docking simulations and surface plasmon resonance (SPR) assays. In vivo studies using CSC-enriched allografts and a paclitaxel-resistant metastatic model were conducted to understand translational relevance.

**Results:**

PITA’s direct inhibition of Mcl-1 enabled potent suppression of TNBC cells by selectively enhancing mitochondrial ROS production, reducing mitochondrial membrane potential, and depleting ATP content, triggering caspase-mediated apoptosis. PITA effectively targeted CSC-like subpopulations, marked by high ALDH1 activity and the CD44^high^/CD24^low^ phenotype. By downregulating p-glycoprotein and Mcl-1/Bcl-2 signaling, PITA was also effective at counteracting paclitaxel resistance, and disrupted AKT/STAT3 survival pathways. PITA significantly inhibited the growth of TNBC patient-derived tumor organoids (PDTOs). Furthermore, its combination with paclitaxel exhibited a synergistic effect on TNBC organoid growth inhibition. In vivo, PITA exhibited potent anti-tumorigenic and anti-metastatic effects, significantly reducing tumor growth and lung metastasis in TNBC allograft models without overt toxicity.

**Conclusion:**

PITA’s inhibition of Mcl-1 represents a novel mechanism to address treatment-refractory metastatic TNBC. Further assessment of PITA’s therapeutic potential is warranted.

**Supplementary Information:**

The online version contains supplementary material available at 10.1186/s40164-025-00716-6.

## Introduction

 Triple-negative breast cancer (TNBC) is the most deadly and aggressive form of breast cancer, accounting for 10–20% of all cases [1, 2]. The five-year overall survival rate is 81%, but for those diagnosed with stage IV, it decreases significantly to 11% [3, 4]. Approximately 46% of TNBC patients develop distant metastases, with a median survival time of only 13.3 months [5]. The absence of estrogen receptor (ER), progesterone receptor (PR), and human epidermal growth factor receptor 2 (HER2) expression in TNBC represents a significant obstacle for the development of targeted therapies and current treatment options involve chemotherapies such as paclitaxel and doxorubicin [6]. TNBC tumors are typically heterogeneous, ultimately limiting the effectiveness of pharmacological interventions [7].

Major mechanisms driving paclitaxel resistance include transporter-mediated drug efflux, tubulin mutations, microtubule alterations, the presence of cancer stem cells (CSCs), and the persistent activation of survival pathways such as PI3K/AKT, NF-kB, and JAK/STAT3 [8, 9]. Notably, while paclitaxel reduces overall tumor burden, it often fails to eliminate dormant CSCs, leading to chemoresistance and recurrence [10]. Breast cancer stem cells (BCSCs), marked by the CD24^low^/CD44^high^ phenotype and elevated ALDH1 activity, form self-renewing spheroids and drive tumor initiation, proliferation, motility, angiogenesis, and dissemination after chemotherapy regimens [10–12]. To halt disease progression and improve survival, it is crucial to eliminate CSC-like populations within TNBC tumors [13].

Myeloid cell leukemia 1 (Mcl-1) is a member of the anti-apoptotic Bcl-2 family and is frequently subject to somatic alteration in various tumor types [14]. Notably, Mcl-1 is amplified in 54% of TNBC cases after neoadjuvant chemotherapy regimens that include paclitaxel [15]. Mcl-1 plays a pivotal role in regulating mitochondrial dynamics and serves as a barrier against mitochondria-dependent apoptosis [16, 17]. Elevated Mcl-1 expression is positively correlated with higher tumor grade, reduced overall survival, and a poorer prognosis in breast cancer [18, 19]. Furthermore, Mcl-1 is crucial for the maintenance of CSC function, cell migration, and invasion, while its blockade has been shown to reduce CSC-load and motility, thereby suppressing tumor growth in vivo [20, 21]. Given these dynamics, targeting Mcl-1 function represents a possible approach to combat relapse and treat refractory metastatic TNBC.

Pitavastatin (PITA) is a competitive 3-hydroxyl-3-methyl glutaryl coenzyme A (HMG-CoA) reductase inhibitor approved for the treatment of hyperlipidemia, and works by reducing total cholesterol and low-density lipoprotein cholesterol (LDL-C) levels in the blood [22]. Its pharmacological toxicity and safety parameters have been determined in clinical trials [23, 24]. Although previous studies have examined the effect of PITA on cancer cell proliferation, apoptosis, and tumor growth in various types of cancers [25–28], the precise mechanisms responsible for its anticancer action have not been fully elucidated. Here, we report for the first time that PITA is a direct inhibitor of Mcl-1 and targets heterogeneity in TNBC cells via the suppression of CSC-like properties, thereby preventing distant metastasis and counteracting paclitaxel resistance.

## Methods

### Breast cancer cell culture

The human TNBC cell line MDA-MB-231 (PerkinElmer Inc. USA), Hs578T (American Type Culture Collection, ATCC), BT549 and murine mammary carcinoma 4T1-Luc (Japanese Collection of Research Bioresources Cell Bank, Japan) were cultured in MEM or RPMI 1640 (Gibco, MD) supplemented with 10% fetal bovine serum (FBS) and streptomycin-penicillin (100 U/mL) at 37 °C with 5% CO_2_. Emergent resistance to paclitaxel in the 4T1 cell line, named PacR-4T1, was established through continuous induction with stepwise escalating concentrations of paclitaxel, ranging from 140 to 1000 nM over a period of 6 months. All cell lines were passaged for less than 6 months after resuscitation and were used from passages 3 to 20. All cell lines were authenticated by short tandem repeat (STR) profiling by Macrogen Inc. (Seoul, South Korea).

### Statistical analysis

All data were analyzed using GraphPad Prism 9.0 statistical software (San Diego, CA). The results are presented as mean ± SEM of at least three independent experiments. Depending on the experimental design, comparisons were conducted using unpaired Student’s t-tests, one-way or two-way ANOVA, and log-rank tests for survival analyses, with Bonferroni’s post hoc test applied for multiple group comparisons. Statistical significance was defined as *p* < 0.05. In addition to p-values, the practical significance of our findings was evaluated by calculating effect sizes (e.g., Cohen’s d, Hedges’ g, and Eta-squared [η^2^]) and by presenting 95% confidence intervals, particularly for key comparisons in gene expression, cell viability assays, and survival outcomes.

## Materials used (details listed in supplementary information 2)

The detailed methods for Reagents, materials, and antibodies, breast cancer cell culture, cell viability assay, sub-G1 analysis and annexin V/PI assay, aldefluor-positivity assay, CD44^high^/CD24^low^ and CD49f^high^/CD24^high^ staining, RNA extraction and RT-qPCR, immunoblot analysis, immunoprecipitation assay, immunocytochemistry, in vitro mammosphere formation assay, limiting dilution assay, organoid culture and drug treatment, cell sorting and cytological centrifugation, detection of reactive oxygen species (ROS) generation, measurement of mitochondrial membrane potential (∆Ψ m), intracellular adenosine triphosphate (ATP) assay, molecular modeling and docking analysis, surface plasmon resonance (SPR) analysis, allograft in vivo experiments and bioluminescence imaging (BLI), serum biochemistry profiles for biomarkers of liver and renal injury, immunohistochemistry and in-situ localization of apoptosis (TUNEL), MMP-2, MMP-9 and VEGF ELISA assay, wound healing and migration assay, public dataset source and bioinformatics analysis, as well as statistical analysis, are described in the Supplementary Materials and Methods section.

## Results

### Pitavastatin (PITA) directly inhibits Mcl-1

**Fig. 1:**
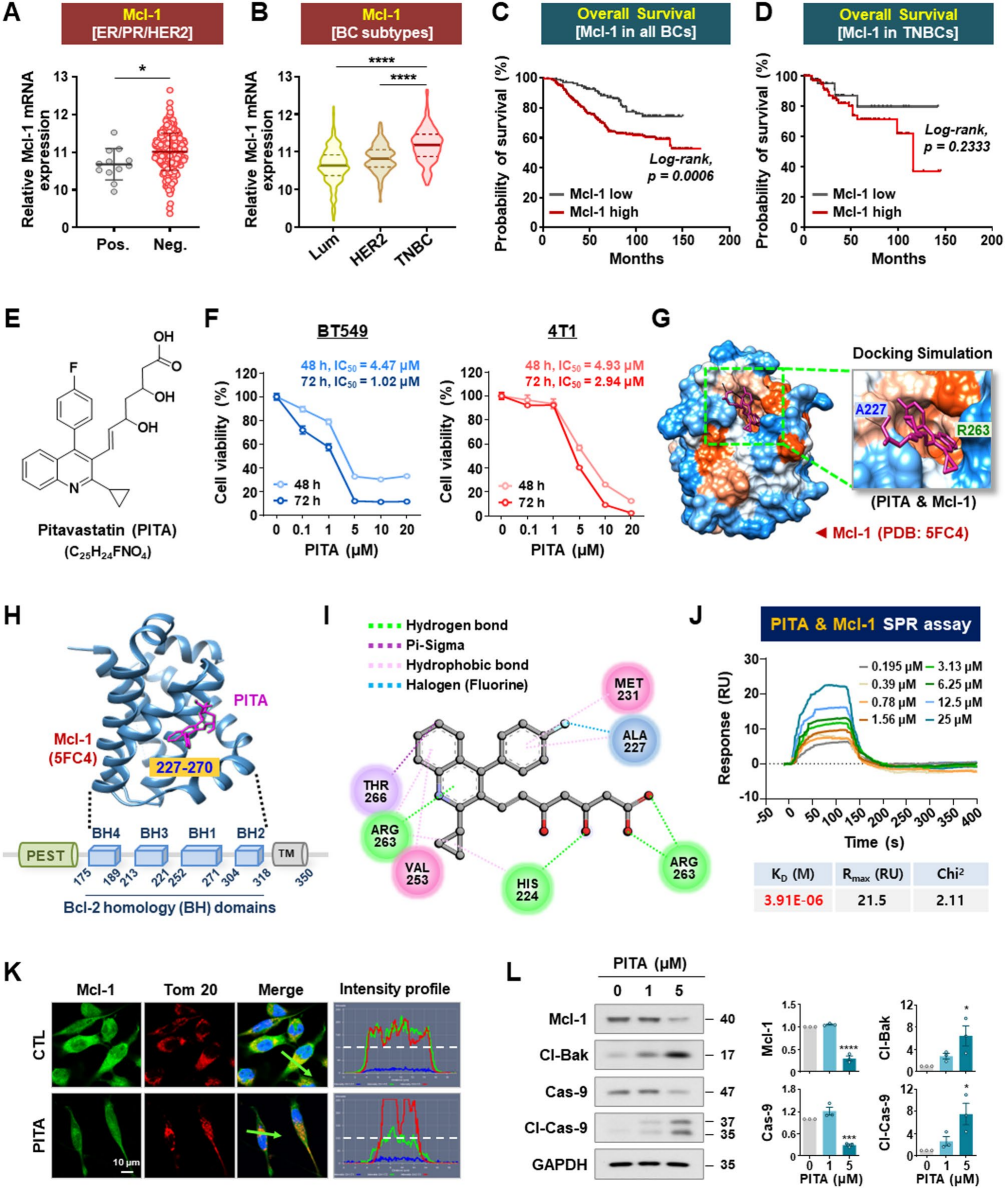
PITA directly targets Mcl-1. **A** Comparison of Mcl-1 mRNA expression between ER/PR/HER2-positive and ER/PR/HER2-negative breast tumor tissue samples using data from the publicly available GENT2 dataset (*p < 0.05). **B** Analysis of mRNA expression for Mcl-1 in subtypes of breast cancer patients [****p < 0.0001, luminal (Lum, *n* = 680), HER2-positive (HER2, *n* = 230), and basal-like/triple-negative subtype (TNBC, *n* = 251)]. **C, D** Kaplan-Meier curves depict overall survival of all breast cancer [**C**, *log-rank*; *p* = 0.0006, Mcl-1-high (*n* = 392), and Mcl-1-low (*n* = 199)] and TNBC patients [**D**, *log-rank*; *p* = 0.2333, Mcl-1-high (*n* = 112) and Mcl-1-low (*n* = 54)] with high and low Mcl-1 mRNA expression using the GENT2 and TCGA database cohort. **E** Chemical structure of PITA. **F** BT549 and 4T1 cells were treated with various concentrations of PITA (0.1–20 µM) or control vehicle (DMSO) for 48 h and 72 h. Cell viability and 50% inhibitory concentrations (IC_50_) were determined by MTS assay. **G–I** In silico molecular docking analysis between PITA and Mcl-1 (PDB: 5FC4). **G** Surface map of lipophilic/hydrophilic properties in the active binding cavity of Mcl-1 (red: hydrophobic, blue: hydrophilic). PITA is shown in a stick model (purple). **H** Binding pose of PITA (pink stick model) in the Bcl-2 homology (BH) domains of Mcl-1 (blue ribbon). **I** 2D diagram analysis of intermolecular interactions between PITA and Mcl-1. Key amino acid residues within the binding pocket are displayed in the ball-and-stick format. Hydrogen bonds (< 5.0 Å) are represented as green dashed lines. **J** Surface plasmon resonance (SPR) binding curves of the interaction between PITA and Mcl-1. The dynamic interaction (equilibrium dissociation constant, K_D_) of PITA with the Mcl-1 protein was calculated to be 3.91 µM. **K** Immunofluorescence analysis of Mcl-1 (green) and Tom 20 (red) with DAPI (nuclei, blue) in BT549 cells following exposure to PITA (5 µM, 48 h). Fluorescence intensity was analyzed using a histogram tool in the Carl Zeiss software. The white dotted straight line indicates 100 intensity units. The y-axis is a range scale covering 0–250 units. **L** Effect of PITA (0–5 µM, 48 h) on Mcl-1, cleaved-Bak, caspase-9, and cleaved-caspase-9 expression in BT549 cells. Quantitative graphs of relative protein contents are shown in the right panels (*p < 0.05). The results are presented as mean ± SEM of at least three independent experiments and analyzed by one-way ANOVA followed by Bonferroni’s post hoc test. CTL, control; PITA, Pitavastatin; Cl-Bak, cleaved Bak; Cas-9, caspase-9: Cl-Cas-9, cleaved caspase-9; Tom 20, translocase of outer mitochondrial membrane 20

Oncogenic Mcl-1 is frequently upregulated in TNBC patients and plays a crucial role in evading apoptosis and diminishing the sensitivity of cancer cells to anticancer agents [29–31]. Analysis of the GENT2 dataset showed that Mcl-1 mRNA expression is low in patients exhibiting concurrent ER/PR/HER2 overexpression (*p* < 0.05, Fig. 1A). Consistent with this observation, the highest levels were observed in TNBC patients (*p* < 0.0001, Fig. 1B). Furthermore, high Mcl-1 mRNA expression is linked to a significantly poorer prognosis compared in breast cancer overall (*p* = 0.0006, Fig. 1C) and TNBC in particular (*p* = 0.2333, Fig. 1D).

PITA is a statin featuring a central quinolinic ring and side chains, including heptenoic acid, fluorophenyl and cyclopropyl groups, that improve pharmacokinetics [32] (Fig. 1E). Despite preclinical studies on PITA’s anticancer efficacy, its mechanism of action has remained unclear [25–28]. We examined the cytotoxic effects of PITA on human BT549, MDA-MB-231 and Hs578T and murine 4T1 TNBC cells, observing that PITA significantly reduced cell viability in both a dose- and time-dependent manner (Fig. 1F and Supplementary Fig. S1). The potential for PITA to bind Mcl-1 was explored through a molecular docking simulation using its crystal structure (PDB: 5FC4). Docking studies revealed that PITA plausibly fits into the BH3-binding groove of Mcl-1 (Fig. 1G-H). The binding pose of PITA with Mcl-1 is extensively stabilized by four hydrogen bonds, one pi-sigma, and two hydrophobic bonds with key amino acid residues Arg263, Thr266, Val253, and His224 in the heptenoic acid group and the quinoline ring of PITA (Fig. 1I). In addition, four hydrophobic interactions and one halogen bond were predicted to form between the fluorophenyl and cyclopropyl moieties of PITA with the active residues of the hydrophobic groove of Mcl-1, including Arg263, Ala227, Met231, and His224 (Fig. 1I). This interaction was further confirmed through surface plasmon resonance (SPR) analysis, which revealed a dose-dependent increase in PITA’s binding affinity for Mcl-1 (Fig. 1J). The equilibrium dissociation constant (K_D_) for PITA binding to Mcl-1 was determined to be 3.91 µM (Fig. 1J), in contrast to the BH3 mimetic Mcl-1 inhibitor AT101 [33] which exhibited lower affinity with a K_D_ of 9.48 µM (Supplementary Fig. S2).

Mcl-1 inhibitors such as S64315, AT101, and AMG-176 are known to increase the expression of Mcl-1 by enhancing the stability of the protein. This stabilization is attributed to a conformational change in the Mcl-1 protein induced by the inhibitor binding [33–35]. Upon treatment with PITA, Mcl-1 levels in BT459 cells initially increased at 6 h, remained stable for up to 12 h, and subsequently decreased by 48 h (Supplementary Fig. S3). Double-fluorescence immunocytochemistry further revealed that Mcl-1 and the mitochondrial marker Tom 20 are colocalized within the mitochondria, as indicated by intensively overlapping fluorescent signals. Mcl-1 expression was markedly diminished following PITA challenge (*p* < 0.05, Fig. 1K-L). Immunoprecipitation using an anti-Mcl-1 antibody confirmed that levels of phospho-ubiquitin (Ser65) increased in the presence of PITA, suggesting that ubiquitination is involved in Mcl-1 degradation (Supplementary Fig. S4A). Immunocytochemical analysis confirmed the co-localization of Mcl-1 and ubiquitin in the cytoplasm following PITA treatment (white arrows, Supplementary Fig. S4B). Mcl-1 inhibition by PITA treatment was accompanied by a marked increase in cleavage of Bak and caspase-9, indicating that PITA triggers dysregulation of mitochondrial function (*p* < 0.05, Fig. 1L and Supplementary Fig. S3).

### PITA induces apoptosis in TNBC cells via mitochondrial dysfunction

**Fig. 2:**
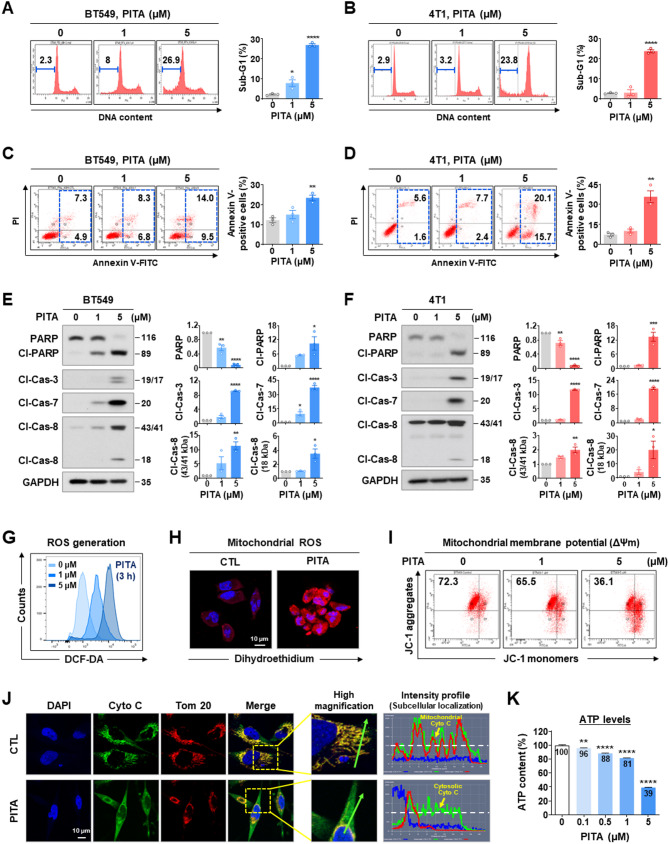
Mitochondrial dysfunction is associated with PITA-induced apoptosis. **A**, **B** Sub-G1 populations in BT549 (**A**, *p < 0.05) and 4T1 cells (**B**, ****p < 0.0001) were analyzed after treatment with PITA (0–5 µM, 48 h). **C-D** Early and late apoptotic cells in the presence or absence of PITA were identified by annexin V/PI staining (**p < 0.01). **E**,** F** Effect of PITA (0–5 µM, 48 h) on PARP, cleaved-PARP, cleaved caspase-3, cleaved caspase-7, and cleaved caspase-8 expression in BT549 (**E**) and 4T1 cells (**F**). Quantitative graphs represent the ratio of protein content (*p < 0.05). **G, H** BT549 cells were treated with PITA (0–5 µM) for 3 h. Intracellular ROS generation was determined by DCF-DA staining (**G**), and mitochondrial ROS production was determined by dihydroethidium (DHE) staining (**H**). **I** Following exposure to PITA (0–5 µM, 48 h), MMP (ΔΨm) in BT549 cells was evaluated by JC-1 assay. **J** BT549 cells were treated with PITA at 5 µM for 24 h and immunostained for cytochrome c (Cyto C, green) and Tom 20 (red, mitochondria) with DAPI (blue, nucleus). The signal intensity of Cyto C (green line) and subcellular localization (yellow arrows) were analyzed using the intensity profile tool. **K** Changes in intracellular ATP levels in BT549 cells following PITA treatment (0–5 µM) for 48 h (**p < 0.01)

Subsequently, we examined early and late apoptosis and the expression levels of apoptosis-related proteins in order to delineate the pathways activated by PITA in vitro. Considering each IC_50_ value (Fig. 1F), a reasonable concentration range of PITA (1–5 µM) was selected for in vitro evaluation. PITA (1–5 µM, 48 h) effectively induced apoptosis in BT549 and 4T1 cells, as evidenced by increased Sub-G1 accumulation (*p* < 0.05, Fig. 2A-B) and a significant rise in both early and late apoptotic cells (*p* < 0.01, Fig. 2C-D, respectively). This apoptotic response was associated with the activation of caspase-3, −7, and − 8, resulting in PARP cleavage in TNBC cells (*p* < 0.05, Fig. 2E-F). PITA increased cellular ROS levels in a dose-dependent manner (Fig. 2G). Confocal imaging analysis using dihydroethidium (DHE), a mitochondrial ROS detector that emits red fluorescence, revealed that cellular ROS production predominantly increased in mitochondria (Fig. 2H and Supplementary Fig. S5). To assess if these mitochondrial alterations trigger cytochrome c release, we conducted double-immunocytochemistry for cytochrome c and Tom 20, a mitochondrial marker. Green signal intensity indicated predominant cytochrome c release from mitochondria to the cytoplasm (Fig. 2J). Inhibition of Mcl-1 by PITA promoted the accumulation of active Bak at the mitochondrial outer membrane, triggering the release of cytochrome c and mitochondrial outer membrane permeability (MOMP), resulting in apoptosome formation. Depletion of ATP content following PITA stimulation appears to result from the loss of MMP and mitochondrial dysfunction (*p* < 0.01, Fig. 2I and K).

### Mcl-1 is upregulated in CSC-like subpopulations and PITA effectively eliminates these cells

**Fig. 3:**
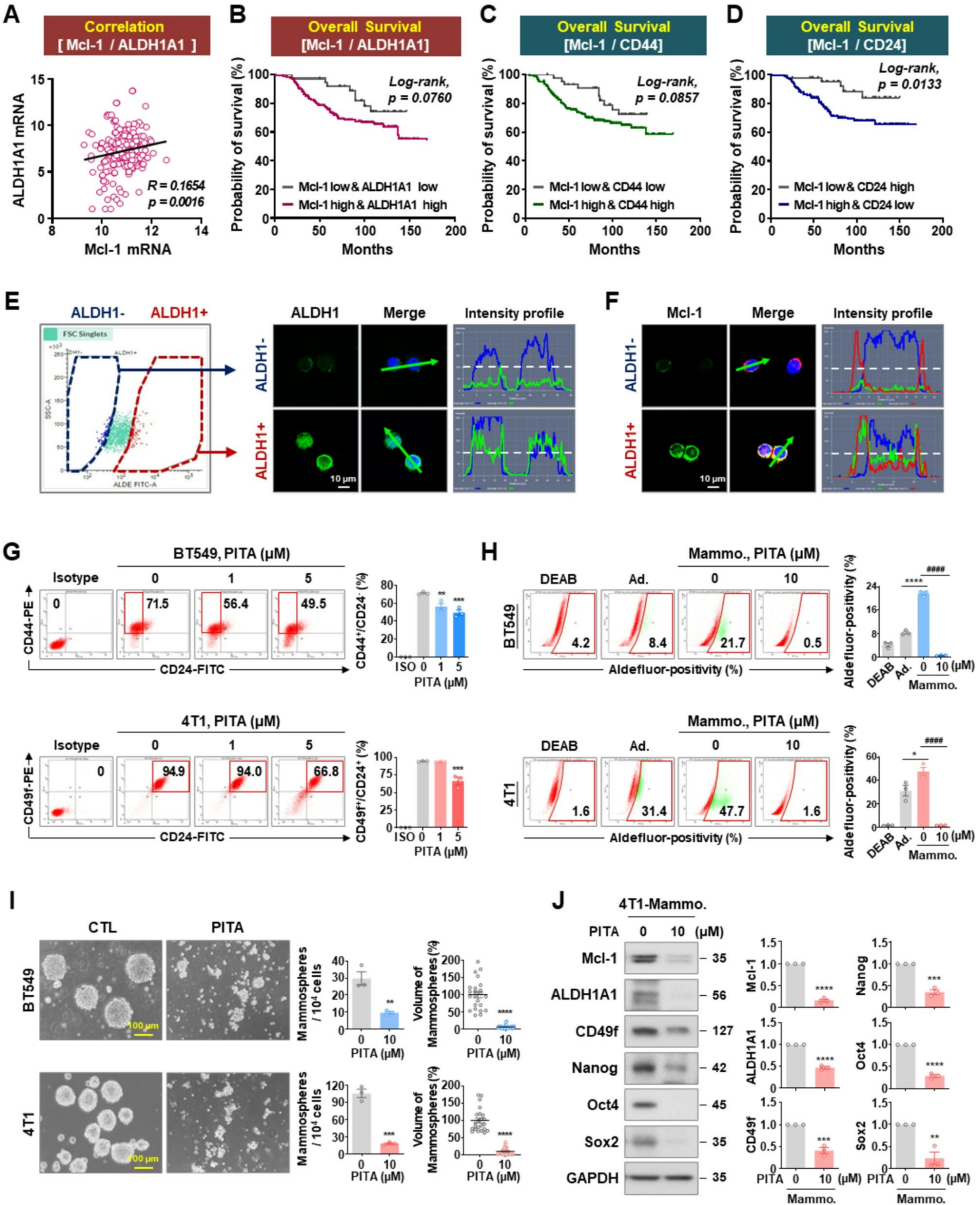
PITA impairs CSC hallmarks. **A** Correlation between Mcl-1 and ALDH1A1 mRNA expression (*p* = 0.0016) in breast cancer patients. The co-expression score between two genes was calculated using Pearson’s correlation coefficient (R). **B-D** Kaplan-Meier curves depict overall survival according to mRNA levels between Mcl-1 and either ALDH1A1 [**B**, *log-rank*; *p* = 0.0760, Mcl-1-low/ALDH1A1-low (*n* = 78) and Mcl-1-high/ALDH1A1–high (*n* = 244)], CD44 [**C**, *log-rank*; *p* = 0.0857, Mcl-1-low/CD44-low (*n* = 87) and Mcl-1-high/CD44–high (*n* = 216)] or CD24 [**D**, *log-rank*; *p* = 0.0133, Mcl-1-low/CD24-high (*n* = 81) and Mcl-1-high/CD24–low (*n* = 183)]. **E-F** ALDH1-positive (+) or ALDH1-negative (-) cells were sorted from dissociated 4T1 mammospheres and immunostained for ALDH1 (**E**, green) and Mcl-1 (**F**, green), with DAPI (blue) and Tom 20 (red). **G-J** Effect of PITA on CSC-like properties in TNBC cells. **G** BT549 and 4T1 cells were treated with PITA (0–5 µM, 48 h). CD44^high^/CD24^low^ and CD49f^high^/CD24^high^ populations were determined by flow cytometry. Quantitative graphs represent subpopulation percentages (**p < 0.01). **H** Aldefluor-positivity in BT549- and 4T1-mammospheres was assessed following exposure to PITA (0–10 µM, 3 days). Quantitative graphs for Aldefluor-positive cells are shown (Ad. vs. Mammo.; *p < 0.05, controls vs. PITA-treated mammospheres; ####*p* < 0.0001, *n* = 3). **I** BT549 (1 × 10^6^ cells/mL) and 4T1 (3 × 10^5^ cells/mL) were cultured in serum-free suspension conditions for 2 days and subsequently treated with PITA (10 µM) or control vehicle for 3 days. The numbers and volumes of mammospheres were quantified by optical microscopy (**p < 0.01). **J** Changes in the expression of Mcl-1, ALDH1A1, CD49f, Nanog, Oct4, and Sox2 following exposure to PITA (0–10 µM, 3 days) in 4T1-mammospheres (**p < 0.01)

Cancer stem cells (CSCs), defined by high ALDH1 activity and distinct markers such as CD44^high^/CD24^low^, are key drivers of tumor initiation, invasion, and metastasis [36, 37]. We analyzed gene correlations between Mcl-1 and ALDH1A1 in TNBC patients using a public dataset, finding a significant positive relationship (*p* = 0.0016, Fig. 3A). Kaplan-Meier survival analysis indicated that overall survival was worse in breast cancer patients who exhibited high levels of both Mcl-1 and ALDH1A1 compared to those with low expression of these genes (*p* = 0.0760, Fig. 3B). Furthermore, patients with high Mcl-1 expression and either higher CD44 or lower CD24 expression had a significantly lower overall survival rate (Fig. 3C-D, *p* = 0.0857 and *p* = 0.0133, respectively).

To confirm the relevance of our in silico genomic analysis, we isolated ALDH1-positive cells from 4T1 mammospheres with self-renewal capacity to determine Mcl-1 upregulation (Fig. 3E). Elevated Mcl-1 expression was present in ALDH1-positive cells compared to their ALDH1-negative counterparts (Fig. 3F). We next examine whether PITA affects CSC-like properties by assessing stem cell surface markers (human CD44^high^/CD24^low^ and murine CD24^high^/CD49f^high^), the progenitor marker ALDH1, and in vitro mammosphere-forming ability. The stem-like populations of CD44^high^/CD24^low^ in the BT549 cells and CD24^high^/CD49f^high^ in 4T1 cells were significantly diminished after exposure to PITA (1–5 µM) for 48 h (*p* < 0.01, Fig. 3G). ALDH1 activity is markedly increased in both human and murine mammospheres compared to their adherent BT549 and 4T1 counterparts, yet PITA treatment strongly abolished this activity (*p* < 0.05, Fig. 3H).

Treatment with PITA led to the dissociation and apoptosis of mammospheres, evidenced by a significant decrease in both the number and volume of mammospheres in BT549 and 4T1 cells (*p* < 0.01, Fig. 3I). This response correlated with marked downregulation of Mcl-1 and substantial reductions in the expression of CSC markers (ALDH1A1, CD49f) and core pluripotency regulators, including Oct4, Nanog, and Sox2 (*p* < 0.01, Fig. 3J). Notably, increased cleavage of caspase-3 and PARP was observed in these stem-enriched populations, suggesting that PITA effectively targets and kills CSCs (Supplementary Fig. S6).

### PITA inhibits the growth of TNBC organoids and CSC-enriched allograft tumors

**Fig. 4:**
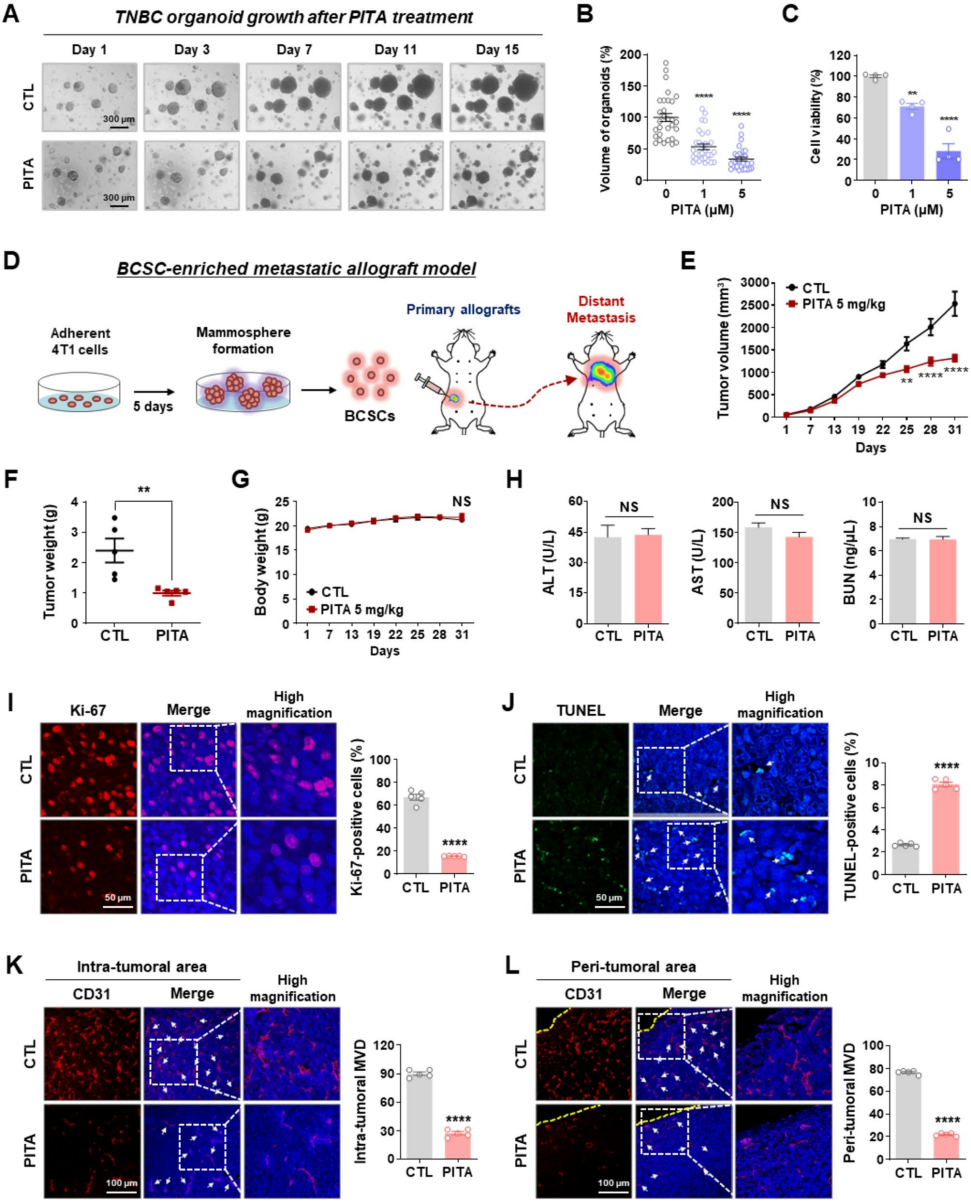
PITA suppresses tumor growth in 4T1-mammosphere-derived allografts.**A-C** Effect of PITA on TNBC organoid growth. **A** Representative images showing the growth of TNBC organoids from day 1 to day 15 after treatment with 5 µM PITA. **B** Quantification of TNBC organoid volume after 15 days of treatment with PITA (0–5 µM), measured using optical microscopy (****p < 0.0001). **C** Organoid cell viability assessed using the CellTiter-Glo 3D assay (**p < 0.01). **D-H** Effect of PITA on tumor growth in vivo. **D** 1 × 10^5^ cells dissociated from 4T1 mammospheres were orthotopically injected into the fourth mammary fat pad of BALB/c female mice. Following exposure to PITA (5 mg/kg, every other day) or a control vehicle for 31 days (*n* = 5 per group), tumor growth (**E**, **p < 0.01), tumor weight (**F**, **p < 0.01), and body weight (**G**, NS, not significant) were evaluated. **H** Influence of PITA on serum biochemical markers of liver and kidney injury. There were no significant changes in ALT, AST, and BUN (NS). **I-J** Effect of PITA on Ki-67 expression and apoptosis in vivo. Tumor tissue sections were immunostained for Ki-67 (**I**, red) with DAPI (blue), and the percentage of Ki-67 positive cells was quantified (****p < 0.0001). Apoptosis induced by PITA in allograft tumors was determined by TUNEL assay (**J**, ****p < 0.0001). **K-L** Impact of PITA on tumor angiogenesis. Tumor tissues were immunostained for CD31 (red) and DAPI (blue). Microvessel density (MVD) was quantified in the intra-tumoral (**K**, ****p < 0.0001) and peri-tumoral areas (**L**, ****p < 0.0001)

To assess the therapeutic efficacy of PITA, TNBC patient-derived tumor organoids (PDTOs) were cultured in a medium that mimics the tumor extracellular matrix. After three days, organoids were treated with PITA (0–5 µM) and monitored daily for 15 days, with identical regions imaged for comparison with their control counterparts (Fig. 4A). By day 15, organoid volume in the PITA-treated groups was significantly reduced in a dose-dependent manner (*p* < 0.0001, Fig. 4B). At the study endpoint, organoid cell viability was assessed using the CellTiter-Glo 3D assay to quantify ATP levels, revealing a significant reduction consistent with the decrease in organoid volume (*p* < 0.01, Fig. 4C). To explore the physiological relevance of our in vitro findings, we generated a tumorigenic and metastatic CSC-inoculated allograft model to evaluate the impact of PITA on tumor growth and dissemination (Fig. 4D). PITA (5 mg/kg, every other day) or control solvent was administered when tumor volumes reached approximately 50 mm^3^. Over 25 days, PITA significantly reduced tumor growth (*p* < 0.01, Fig. 4E) and burden (*p* < 0.01, Fig. 4F), with no notable effect on mean body weight (NS; not significant, Fig. 4G). To evaluate the potential organ toxicity of PITA, we conducted assays for aspartate aminotransferase (AST), alanine aminotransferase (ALT), and blood urea nitrogen (BUN) using serum samples from mice. There were no significant changes in AST and ALT levels, indicators of liver function, or BUN levels, markers of renal function (NS, Fig. 4H).

Subsequently, we observed that PITA’s impairing effects on tumor growth were accompanied by a marked reduction in Ki-67 index (*p* < 0.0001, Fig. 4I) and enhancement of apoptosis in tumor tissues (*p* < 0.0001, Fig. 4J), as indicated by increased caspase-3 cleavage (*p* < 0.0001, Supplementary Fig. S7). To assess the impact of PITA on angiogenesis, levels of vascular endothelial growth factor (VEGF) in the circulating serum of mice and microvessel density (MVD) within the tumors were evaluated. Compared to healthy mice, a significant increase in blood VEGF levels was observed during metastasis from primary tumors in CSC-inoculated allografts. Notably, the elevated VEGF levels in these allografts were significantly diminished following PITA treatment (*p* < 0.001, Supplementary Fig. S8). Concurrently, the number of CD31-positive microvessels in both the intra-tumoral (*p* < 0.0001, Fig. 4K) and peri-tumoral (*p* < 0.0001, Fig. 4L) regions was significantly reduced following PITA administration.

### PITA suppresses metastasis from the primary TNBC lesion

**Fig. 5:**
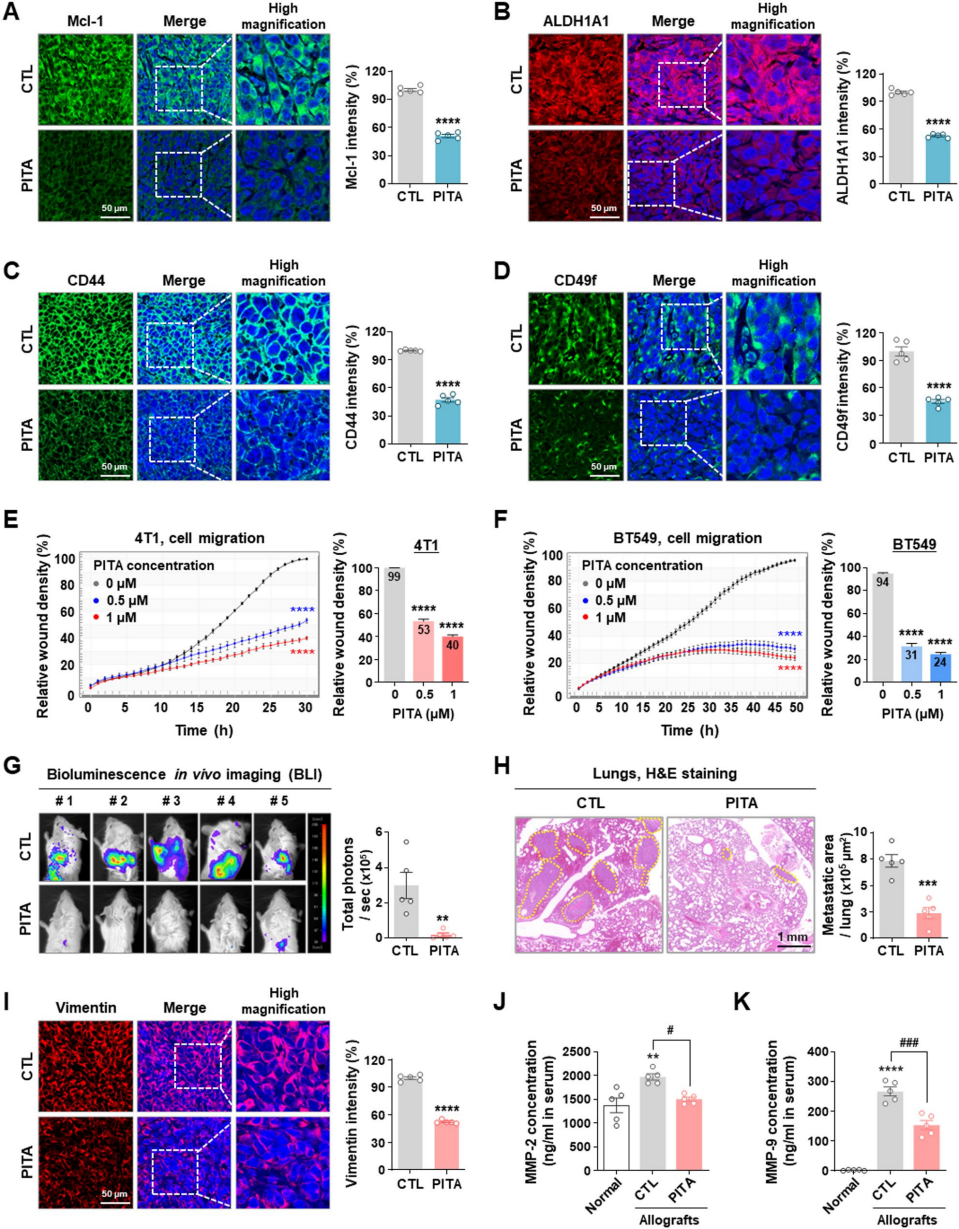
PITA suppresses TNBC metastasis. **A–D** Immunohistochemical analyses for Mcl-1 (**A**), ALDH1A1 (**B**), CD44 (**C**), and CD49f (**D**) in allograft tumors with fluorescence intensities quantified (****p < 0.0001). **E, F** Effect of PITA on cell migration. After treatment with PITA (0–1 µM) in 4T1 (**E**, 30 h) and BT549 cells (**F**, 48 h), kinetic analysis of cell migration (****p < 0.0001). Quantitative graphs represent relative wound density at 30 h and 48 h, respectively (right panels, ****p < 0.0001). **G** Impact of PITA on distant metastasis in vivo. Representative BLI of metastasis in the CTL- and PITA-treated groups (**p < 0.01). **H** Hematoxylin and eosin (H&E) staining of lung sections from CTL- and PITA-treated mice. Yellow-dotted regions indicate metastatic lesions and areas were quantified (***p < 0.001). **I** Immunohistochemical analysis of vimentin in allograft tumors (****p < 0.0001). **J-K** Impact of PITA on serum levels of MMP-2 and MMP-9 in vivo. Serum levels of MMP-2 (**J**) and MMP-9 (**K**) were measured by ELISA in the CTL- and PITA-treated groups (normal mice vs. control; **p < 0.01, control vs. PITA-treated group; #*p* < 0.05)

In silico docking simulation and SPR analysis confirmed that PITA is an inhibitor of Mcl-1, while in vivo studies revealed significant downregulation of Mcl-1 expression in allograft tumors following treatment with PITA (*p* < 0.0001, Fig. 5A). Our in vitro studies revealed that PITA targets CSC hallmarks, indicated by decreased ALDH1A1 activity and reductions in the CD44^high^/CD24^low^ and CD24^high^/CD49f^high^ stem-like subpopulations. Immunohistochemical analyses for the mammary stem cell markers ALDH1A1, CD44, and integrin alpha 6 (CD49f) on allograft tumors revealed significant downregulation of ALDH1A1 (*p* < 0.0001, Fig. 5B), CD44 (*p* < 0.0001, Fig. 5C), and CD49f (*p* < 0.0001, Fig. 5D) in PITA-treated mice. We next assessed the impact of PITA on metastasis in CSC-enriched allografts. Kinetic analysis revealed that PITA significantly inhibited cell migration in BT549 and 4T1 cells in a dose-dependent manner (*p* < 0.0001, Fig. 5E-F). Consistently, PITA treatment reduced lung metastasis, as demonstrated by notable decreases in bioluminescence intensity (*p* < 0.01, Fig. 5G) and the number of metastatic lung nodules (*p* < 0.001, Fig. 5H). PITA administration also significantly downregulated phospho-STAT3 and vimentin expression in tumor tissues derived from allografts, potentially inhibiting tumor cell propagation (*p* < 0.0001, Fig. 5I and Supplementary Fig. S9). Additionally, serum levels of matrix metalloproteinases MMP-2 and MMP-9, which are critical for tumor cell migration, angiogenesis, and metastasis [38, 39], were markedly higher in metastatic control mice compared to normal mice. These levels were significantly reduced in the PITA-treated group (*p* < 0.05, Fig. 5J-K).

### PITA overcomes paclitaxel resistance in TNBC

**Fig. 6:**
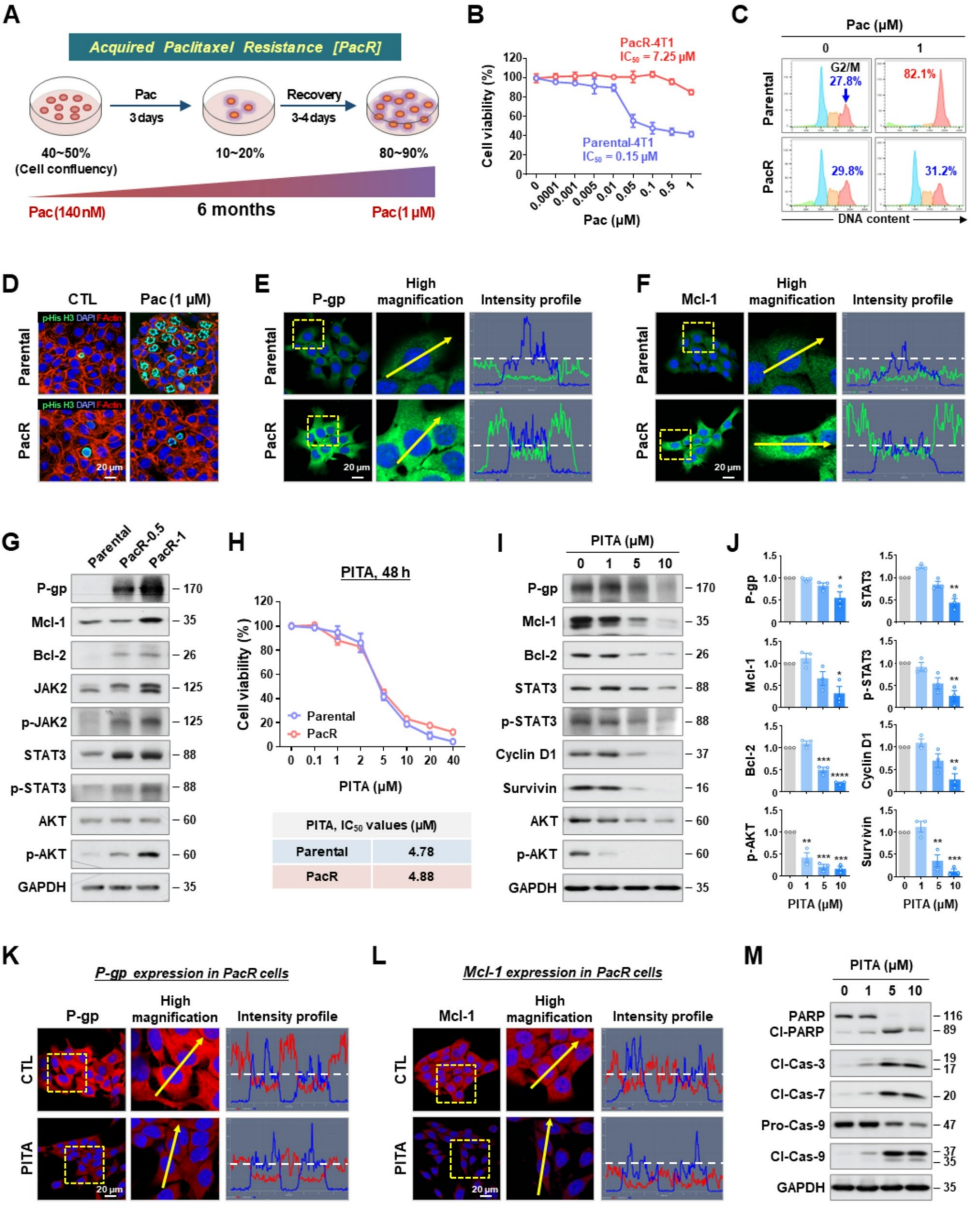
PITA exerts potent antiproliferative effects in paclitaxel-resistant TNBC cells. **A** Paclitaxel‑resistant 4T1 (PacR-4T1) cells were established by dose-escalating treatment of 140 nM to 1 µM of paclitaxel for 6 months. **B–G** Characterization of acquired paclitaxel resistance in 4T1 cells. **B** Parental-4T1 and PacR-4T1 cells were treated with Pac (0.0001–1.0001 µM) for 48 h. Cell viability and IC_50_ values were analyzed by MTS assay. **C** Cell cycle distribution in parental- and PacR-4T1 cells after treatment with Pac (1 µM, 12 h). Representative histogram images show the proportion of cells in each phase of the cell cycle (G2/M fractions, blue arrows). **D** Immunofluorescence analysis for p-His H3 (green), F-actin (red), and DAPI (blue) in parental- and PacR-4T1 cells following exposure to Pac (1 µM, 12 h). **E, F** Immunocytochemical analysis for P-gp (**E**) and Mcl-1 (**F)** in parental-4T1 and PacR-4T1 cells. **G** Changes in the expression of P-gp, Mcl-1, Bcl-2, JAK2, p-JAK2, STAT3, p-STAT3, AKT, and p-AKT in parental- and PacR-4T1 cells (PacR-0.5, resistant to 0.5 µM of Pac; PacR-1, resistant to 1 µM of Pac). **H** Parental- and PacR-4T1 cells were treated with PITA (0.1–40 µM) for 48 h. Cell viability and IC_50_ values were determined by MTS assay. **I** Effect of PITA (0–10 µM, 48 h) on the expression of P-gp, Mcl-1, Bcl-2, STAT3, p-STAT3, cyclin D1, survivin, AKT, and p-AKT in PacR-4T1 cells. **J** Quantitative graphs represent relative protein content (*p < 0.05). **K**, **L** Immunofluorescence analysis of P-gp (**K**) and Mcl-1 (**L**) in PacR-4T1 cells following exposure to PITA (10 µM, 24 h). **M** Effect of PITA (0–10 µM, 48 h) on the expression of PARP, cleaved-PARP, cleaved caspase-3, cleaved caspase-7, caspase-9, and cleaved caspase-9 in PacR-4T1 cells

To establish a paclitaxel-resistant cell line, 4T1 cells were treated with increasing concentrations of paclitaxel starting from the IC_30_ value of 140 nM. The cells were cultured for 6 months with alternating fresh medium and paclitaxel until they retained viability and the ability to divide at a maximum concentration of 1 µM paclitaxel. Cell viability was periodically monitored using an MTS assay, confirming resistance with 84.5% viability at 1 µM paclitaxel (Fig. 6A). The MTS assay revealed that the PacR-4T1 cells (IC_50_, 7.25 µM) were 48-fold more resistant to paclitaxel than the parental-4T1 cells (IC_50_, 0.15 µM) (Fig. 6B). Treatment with paclitaxel (1 µM) significantly increased the G2/M phase cell population to approximately 82.1% in 4T1 cells. In contrast, in PacR-4T1 cells, the accumulation of G2/M phase was limited to only 31.2% following paclitaxel treatment, which was comparable to the 29.8% observed in the untreated control group (Fig. 6C and Supplementary Fig. S10). This phenomenon was further supported by the expression of phospho-histone H3 (Ser10) (p-His H3), a marker of G2/M phase. In 4T1 cells, paclitaxel treatment markedly increased the levels of p-His H3, indicating abnormal chromosome segregation. Meanwhile, in PacR-4T1 cells, the expression remained comparable to that of the control (Fig. 6D and Supplementary Fig. S11).

We observed that P-glycoprotein (P-gp), a major obstacle of paclitaxel resistance is predominantly overexpressed in the plasma membrane of PacR-4T1 cells (Fig. 6E). Western blot analysis revealed that P-gp expression increased with higher paclitaxel concentrations in 4T1 cell lines that developed resistance to 0.5 µM and 1 µM paclitaxel (Fig. 6G). In the PacR-4T1 cells that developed paclitaxel resistance, both phospho-JAK2 (Tyr1007/1008) and phospho-STAT3 (Tyr705) were upregulated (Fig. 6G). Additionally, activation of STAT3 coincided with enhanced expression of CSC-related factors, including CD44, ALDH1A1, Nanog, Oct4, and Sox2. Concurrently, the protein content of vimentin, a marker of the mesenchymal phenotype, also showed a significant increase (Supplementary Fig. S12). Fluorescence and intensity signals indicated marked upregulation of Mcl-1 in PacR-4T1 cells (Fig. 6F). Western blot analysis showed that Mcl-1 expression increased in cells resistant to higher concentrations of paclitaxel (Fig. 6G). Consistently, analysis of the GEO dataset GSE162187 showed significant upregulation of MCL1 transcripts in taxane-resistant breast cancer patients compared with taxane-sensitive patients (Supplementary Fig. S13), supporting a correlation between Mcl-1 induction and taxane resistance.

We assessed the impact of PITA on cell viability in PacR-4T1 cells, which exhibit a 48-fold higher resistance to paclitaxel compared to the parental-4T1 cells. PITA reduced cell viability in both paclitaxel-sensitive and paclitaxel-resistant cells in a dose-dependent manner. The IC_50_ values of PITA in parental-4T1 cells and PacR-4T1 cells were 4.78 µM and 4.88 µM, respectively, indicating no substantial difference in drug sensitivity between these two cell lines (Fig. 6H). The comparative effects of PITA and Mcl-1 inhibitors, S63845, and AT101 on cell viability were evaluated in TNBC cells. In BT549 and 4T1 cells, PITA exhibited the lowest IC_50_ values (1.087 and 2.962 µM), whereas S63845 (11.03 and 12.68 µM) and AT101 (6.129 and 5.582 µM) displayed 4- to 10-fold and 2- to 5-fold higher values, respectively. In PacR-4T1 cells, PITA retained potent activity (IC_50_ = 3.962 µM), approximately 15-fold lower than S63845 (59.27 µM) and 1.38-fold lower than AT101 (5.482 µM), highlighting its superior efficacy in resistant cells (Supplementary Fig. S14A-B). Molecular docking simulations predicted that PITA binds Mcl-1 (PDB: 5FC4) with a binding affinity of − 9.462 kcal/mol and an interaction energy of − 39.8 kcal/mol (Supplementary Fig. S14C). In comparison, the calculated binding affinities of S63845 and AT101 were − 9.394 kcal/mol and − 8.938 kcal/mol, respectively. These results indicate that PITA exhibits slightly stronger binding affinity to Mcl-1 than S63845 and markedly higher affinity than AT101 (Supplementary Fig. S14C).

The antiproliferative effects of PITA were associated with the downregulation of MDR1 and reduced expression of Mcl-1/Bcl-2, along with the dysregulation of key survival pathways, including AKT and STAT3 signaling (*p* < 0.05, Fig. 6I-L). Activation of the STAT3 signaling pathway during the acquisition of paclitaxel resistance was significantly suppressed by PITA with a concomitant decrease in survivin and cyclin D1 levels (*p* < 0.05, Fig. 6I-J). In the PacR-4T1 cell line, which is characterized by the overexpression of both Mcl-1 and Bcl-2, treatment with PITA led to a marked reduction in the levels of these proteins, as determined by immunoblotting and immunofluorescence analysis (Fig. 6G-L). PITA-induced apoptosis involved the activation of caspases-3, −7, and − 9 and enhanced PARP cleavage, indicative of intrinsic mitochondria-dependent apoptosis in PacR-4T1 cells (Fig. 6M). Moreover, PITA treatment markedly decreased vimentin mRNA expression (*p* < 0.01, Supplementary Fig. S15A) and significantly impaired the migratory capacity of resistant cells (*p* < 0.001, Supplementary Fig. S15B).

### PITA suppresses metastatic colony formation in paclitaxel-resistant TNBC

**Fig. 7:**
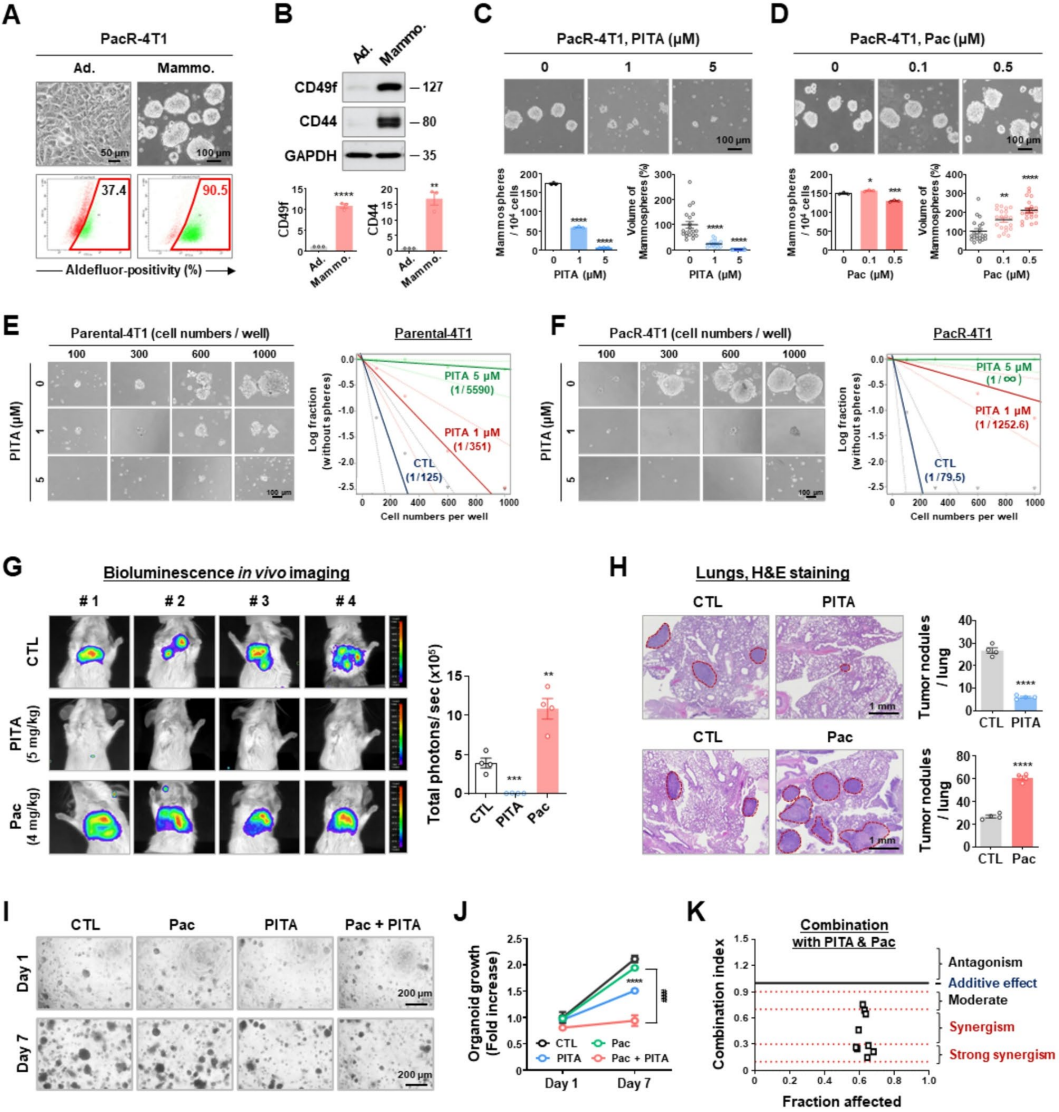
PITA suppresses metastatic potential in paclitaxel-resistant TNBC cells. **A** PacR-4T1 cells (3 × 10^5^ cells/mL) were cultured in normal culture medium or serum-free suspension conditions for 5 days. Aldefluor-positivity was determined by flow cytometry. **B** Changes in the expression of CD49f and CD44 in PacR-4T1 mammospheres (**p < 0.01). **C-D** Comparison of mammosphere-forming ability in PacR-4T1 cells following exposure to PITA (**C**, 0–5 µM) or Pac (**D**, 0–0.5.5 µM) for 5 days (*p < 0.05). **E-F** Limiting dilution assay in parental- (**E**) and PacR-4T1 cells (**F**). Mammospheres were grown for 8 days at different cell seeding densities (100–1000 cells per well). ELDA (extreme limiting dilution analysis) plots show CSC frequency estimates. Scale bar = 100 μm. **G-H** Impact of PITA on lung colonization of PacR-4T1 cells in vivo. 1 × 10^5^ cells derived from PacR-4T1 mammospheres were inoculated into the tail vein of female BALB/c mice, followed by an immediate intravenous administration of PITA (5 mg/kg), Pac (4 mg/kg) or control vehicle. **G** Representative BLI of lung metastasis in control, PITA- or Pac-treated mice (**p < 0.01, *n* = 4). **H** H&E staining analysis of lung sections. The red dotted areas indicate metastatic lesions in the lungs. The number of metastatic nodules was quantified (****p < 0.0001). **I-K** Synergistic effect of combined PITA and paclitaxel treatment on TNBC organoid growth inhibition. **I** Representative images depicting TNBC organoid growth on days 1 and 7 following treatment with PITA (5 µM), paclitaxel (5 nM), or their combination. **J** Organoid growth rate was evaluated using CellTiter-Glo 3D analysis on days 1 and 7 following a 7-day treatment with PITA and/or paclitaxel (****p < 0.0001; PITA only vs. control; NS, Pac only vs. control; ####*p* < 0.001, Pac only vs. combination). **K** The combination index (CI) was calculated to evaluate the drug-drug synergy of various dose combinations of PITA (10–40 µM, 7 days) with paclitaxel (10–40 nM, 7 days) in TNBC organoids. CI values were generated using the CompuSyn software to quantify drug interactions, where CI < 1 indicates synergism, CI = 1 indicates an additive effect, and CI > 1 indicates antagonism

To investigate whether PITA suppresses CSC-like characteristics associated with paclitaxel resistance, we first compared the expression levels of CSC markers in adherent PacR-4T1 cells and PacR-4T1 mammospheres in a 3D suspension culture (Fig. 7A). Notably, mammospheres originating from PacR-4T1 cells displayed a substantially higher aldehyde-positive population (over 90%) compared to the adherent cells (37.4%), coinciding with markedly elevated levels of CD49f and CD44 (*p* < 0.01, Fig. 7B). Paclitaxel had a minimal effect on the number of mammospheres but significantly increased their volumes, even at a high concentration of 0.5 µM (*p* < 0.05, Fig. 7D). PITA impaired this mammosphere-forming ability, as demonstrated by a significant reduction in both the number and volume of mammospheres with paclitaxel resistance characteristics (*p* < 0.0001, Fig. 7C). To functionally validate the effect of PITA on CSC elimination, we performed limiting dilution assays (LDA) in parental 4T1 and PacR-4T1 cells under mammosphere-forming conditions. In parental 4T1 cells, CSC frequency was 1/125 in controls, reduced to 1/351 with 1 µM PITA, and markedly decreased to 1/5590 with 5 µM PITA (Fig. 7E). In PacR-4T1 cells, CSC frequency was higher than in parental cells (1/79 in controls) but was reduced to 1/1252 with 1 µM PITA and completely eliminated with 5 µM PITA (Fig. 7F and Supplementary Fig. S16). These results demonstrate that PacR-4T1 cells are enriched in CSC populations compared with parental cells, and that PITA effectively eliminates CSCs, with particularly strong efficacy in the paclitaxel-resistant context.

In an in vivo metastasis model [40], the effect of PITA on the dissemination and pulmonary colonization of CSCs resistant to paclitaxel was assessed. Dissociated cells from PacR-4T1 mammospheres were intravenously injected into the tail vein of female BALB/c mice, and a single intravenous dose of either 5 mg/kg PITA or 4 mg/kg paclitaxel was administered. BLI imaging analysis performed 28 days post-injection demonstrated a significant reduction in luminescence intensity within the pulmonary regions of the mice treated with PITA, suggesting substantial inhibition of lung colonization by PacR-4T1 spheroid cells (*p* < 0.01, Fig. 7G). In contrast, the paclitaxel-treated group exhibited a marked increase in distant metastasis (Fig. 7G). Representative hematoxylin and eosin (H&E) staining corroborated the findings that PITA administration led to a reduction in the number of lung colonies, while extensive lung colonization was further observed following paclitaxel administration (*p* < 0.0001, Fig. 7H). These observations suggest that the augmentation of CSC-like characteristics induced by paclitaxel may have contributed to the enhanced metastasis. Liver and kidney function tests showed no significant toxicity, except for elevated ALT and AST levels in paclitaxel-treated mice, likely due to liver metastases (*p <* 0.01, Supplementary Fig. S17).

Finally, we evaluated the therapeutic efficacy of combined PITA and paclitaxel therapy in a TNBC organoid model. Although 5 nM paclitaxel alone minimally affected organoid volume, the combination of paclitaxel with 5 µM PITA significantly reduced organoid size (*p* < 0.0001, Fig. 7I-J). Furthermore, an organoid growth rate analysis revealed that PITA alone effectively inhibited organoid growth during 7-day, whereas paclitaxel had limited efficacy (*p* < 0.0001, Fig. 7J). Consistent with these findings, organoid cell viability measurements demonstrated that the combination treatment significantly decreased cell viability compared to either PITA or paclitaxel alone (*p* < 0.0001, Supplementary Fig. S18). The combination index (CI) analysis highlighted that PITA and paclitaxel exerted a strong synergistic effect in TNBC organoids, with CI values consistently below 1 across a range of fraction affected (Fa) values. (Fig. 7K).

## Discussion

Drug repurposing, which identifies new uses for clinically approved drugs, strategically reduces development risk by leveraging the existing data on manufacturing, pharmacokinetics, and toxicity, potentially increasing success rates [41, 42]. PITA is primarily prescribed as an HMG-CoA reductase inhibitor to reduce LDL cholesterol levels; however, beyond its lipid-lowering activity, it exerts multiple pleiotropic effects. These include anti-inflammatory, antioxidant, antiproliferative, and immunomodulatory actions mediated through diverse cellular pathways [43–46]. In the present study, we highlight the potential of PITA to target both bulk tumor cells and CSCs, which are frequently implicated in therapeutic resistance and tumor recurrence in TNBC.

PITA’s unique structure, featuring a central quinolinic ring and side chains such as heptenoic acid, fluorophenyl, and cyclopropyl groups, provides favorable pharmacokinetic properties, including high absorption (80%) and an absolute bioavailability greater than 60%, which is significantly higher compared to other statins (less than 30%) [32, 47]. In mice, the maximum tolerated dose was found to be 75 mg/kg/day, without significant carcinogenic side effects after long-term exposure of 92 weeks. In rats, PITA had no adverse effects on male or female fertility at oral doses of 10 mg/kg/day and 30 mg/kg/day, respectively [48, 49]. In our study, 5 mg/kg of PITA was administered to allografted mice once every other day for 31 days. No significant differences in liver and kidney function were observed, nor were any general toxicity symptoms or histopathological abnormalities detected.

The ability of PITA to bind effectively to the BH3-binding groove of Mcl-1, as confirmed through docking study, is comparable to the selective Mcl-1 inhibitor S64315, which is currently in phase I clinical trials for AML and targets hot-spot sites in Mcl-1, including the P1-P4 pockets and Arg263 [14, 50]. The intrinsic apoptosis cascade is primarily governed by the pro-survival and anti-apoptotic Bcl-2 family members. Mcl-1 plays a critical role in maintaining mitochondrial integrity and preventing apoptosis by blocking the activation of pro-apoptotic Bcl-2 family members, such as Bax and Bak [16, 51, 52]. Notably, inhibition of Mcl-1 by PITA led to significant mitochondrial dysfunction, marked by increased mitochondrial ROS production, and cytochrome c release, ultimately resulting in the activation of the intrinsic apoptotic cascade. This mitochondrial destabilization, driven by Bak cleavage and caspase-9 activation, resulted in caspase-3/−7 activity and apoptosis in TNBC cells, highlighting PITA’s ability to target mitochondrial dynamics for therapeutic effect.

Mcl-1 is indispensable for the survival and self-renewal of human pluripotent stem cells (hPSCs) and is frequently overexpressed in various CSCs, including those in AML and breast cancer [53, 54]. It maintains stem cell pluripotency by regulating mitochondrial dynamics and transcription factors such as Nanog and Oct4 in hepatocellular carcinoma and hPSCs [55–57]. Mcl-1-specific inhibitors disrupt tumor sphere formation, induce apoptosis signaling, and diminish the expression of pluripotency factors including Nanog, Sox2, and KLF4 [56]. Our findings indicate that Mcl-1 is markedly overexpressed in the CSC-enriched population of TNBC and exhibits a significant correlation with ALDH1A1 expression. Elevated levels of both ALDH1A1 and Mcl-1 are associated with poorer patient survival, suggesting that Mcl-1 is a key determinant of CSC fate, promoting tumor aggressiveness and recurrence. Importantly, PITA treatment led to the downregulation of Mcl-1 in CSC-like subpopulations, resulting in decreased mammosphere formation, reduced expression of CSC markers (CD44 and ALDH1A1), and downregulated pluripotency regulators (Nanog, Oct4, and Sox2). CD44^high^/CD24^low^ and CD24^high^/CD49f^high^ phenotypes are associated with mesenchymal-like CSCs, whereas ALDH1 activity defines epithelial-like CSCs with greater clonogenicity and metastatic colonization potential [58]. In line with this, PITA treatment modestly reduced the CD44^high^/CD24^low^ population but strongly inhibited ALDH1 activity, suggesting preferential targeting of epithelial-like CSCs. Together, these results strongly support the notion that PITA effectively targets CSC-driven tumor progression and metastasis in TNBC.

Importantly, CSC subtypes are not fixed but exhibit dynamic plasticity, and this transition is tightly interconnected with epithelial–mesenchymal transition (EMT) programs [59]. EMT not only drives tumor invasion and metastasis but also enriches CSC-like traits and contributes to therapeutic resistance [60]. In our study, PacR-4T1 cells exhibited enrichment of CSC markers, elevated vimentin expression, and increased STAT3 phosphorylation compared with parental cells. Given that STAT3 is a key regulator of CSC maintenance, EMT induction, and tumor recurrence, these findings highlight its central role in resistance [61]. Notably, PITA treatment suppressed STAT3 activation, reduced vimentin mRNA levels, and markedly impaired the migratory ability of resistant cells, indicating that PITA overcomes paclitaxel resistance at least in part through inhibition of the STAT3–EMT axis.

A crucial mechanism underlying paclitaxel resistance is the overexpression of P-glycoprotein (P-gp), which is associated with multidrug resistance. P-gp, primarily located on the cell membrane, actively transports paclitaxel out of cells, substantially reducing its therapeutic efficacy and often leading to treatment failure [62–64]. Developing a pharmacological agent that either inhibits P-gp activity or is not a substrate for it may help overcome drug resistance. A significant association has been observed between P-gp overexpression and STAT3 activation in the context of chemoresistance. Activation of the STAT3/CEBPD axis enhances the expression of MDR1/ABCB1, thereby inducing chemoresistance through the elevation and activation of P-gp [65–68]. Preclinical studies have confirmed that the inhibition of STAT3 suppresses P-gp expression, thereby enhancing drug sensitivity [65, 66]. Furthermore, STAT3 directly upregulates Mcl-1 expression by binding to its promoter. This interaction is pivotal in tumor cell survival and propagation, positioning the STAT3/Mcl-1 axis as a critical target for cancer therapy [20, 69]. Treatment with PITA results in suppression of STAT3 activation and its impact on reducing P-glycoprotein (P-gp) expression suggest that it could enhance sensitivity to paclitaxel and other chemotherapeutic agents.

## Conclusion

**Fig. 8:**
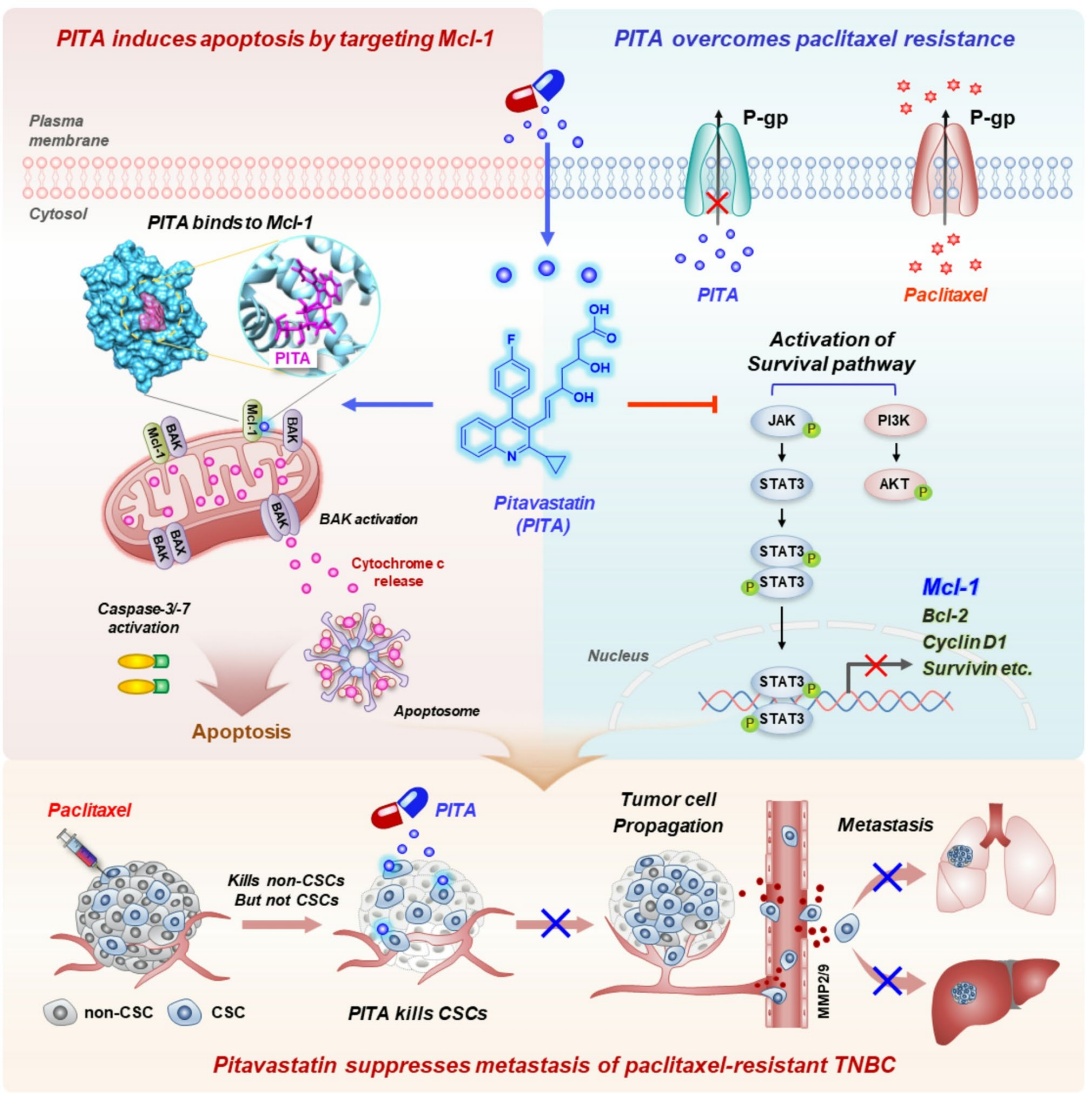
Hypothetical model illustrating the actions of PITA on paclitaxel resistance and TNBC. The widely-used cholesterol-lowering statin pitavastatin represents a novel therapeutic option for TNBC. It binds to the BH3-binding groove within Mcl-1, inducing mitochondrial dysfunction that elevates ROS production and cytochrome c release, triggering caspase-mediated apoptosis. Additionally, PITA counteracts paclitaxel resistance by suppressing P-gp and modulating the STAT3/AKT signaling pathway. By targeting cancer stem-like cells, PITA reduces tumor growth and inhibits the metastatic spread of TNBC. These findings warrant further investigation of pitavastatin’s potential as a targeted therapeutic agent to overcome chemoresistance and treat aggressive forms of TNBC

Several Mcl-1 inhibitors are currently in clinical trials, although none have been approved. Our findings show that PITA acts as a potent Mcl-1 inhibitor with significant anticancer activity against TNBC in vivo. PITA’s inhibition of Mcl-1, disrupts mitochondrial function, enhances ROS production, and triggers mitochondrial-mediated apoptosis. In CSC-enriched tumor models, PITA displays potent anti-tumorigenic and anti-metastatic capacity, effectively targeting CSC-like subpopulations that are typically resistant to conventional therapies and implicated in tumor recurrence and metastasis. This is particularly noteworthy because PITA has the potential to overcome chemotherapy resistance by inhibiting cell viability and pro-survival pathways, including AKT and JAK/STAT3 signaling, resulting in a marked reduction in lung colonization in paclitaxel-resistant TNBC. Importantly, PITA showed greater efficacy than S63845 and AT101, particularly in paclitaxel-resistant TNBC, and significantly inhibited organoid growth, with combination treatment highlighting a synergistic effect with paclitaxel. Given its established safety as an FDA-approved statin and lack of hepatotoxicity or nephrotoxicity, these findings underscore the potential of PITA as a safe and effective therapeutic option. Taken together, our results support PITA as a promising candidate for drug repurposing, particularly in TNBC characterized by high Mcl-1 expression and resistance to conventional chemotherapy (Fig. 8).

## Supplementary Information

Supplementary Material 1

Supplementary Material 2

Supplementary Material 3

## Data Availability

No datasets were generated or analysed during the current study.

## Abbreviations

AKT: Protein kinase B
ALDH1: Aldehyde dehydrogenase 1
ALT: Alanine aminotransferase
AST: Aspartate aminotransferase
Bak: Bcl-2 homologous antagonist/killer
Bcl-2: B-cell lymphoma 2
BUN: Blood urea nitrogen
CSC: Cancer stem cell
CD31: Cluster of differentiation 31
CD44: Cluster of differentiation 44
GENT2: Gene Expression database of Normal and Tumor tissues 2
JAK: Janus tyrosine kinase
Mcl-1: Myeloid cell leukemia 1
MMP: Matrix metalloproteinase
MMP (ΔΨm): Mitochondrial membrane potential
Nanog: Nanog homeobox
Oct4: Octamer-binding transcription factor 4
PDTO: Patient-derived tumor organoid
P-gp: Permeability glycoprotein
ROS: Reactive oxygen species
Sox2: SRY-Box transcription factor 2
STAT3: Signal transducer and activator of transcription 3
TCGA: The Cancer Genome Atlas
Tom 20: Translocase of outer mitochondrial membrane 20
VEGF: Vascular endothelial growth factor

## References

[1] Kumar P, Aggarwal R. An overview of triple-negative breast cancer. Arch Gynecol Obstet. 2016;293:247–69. 26341644 10.1007/s00404-015-3859-y

[2] Li T, Zhang H, Lian M, He Q, Lv M, Zhai L, et al. Global status and attributable risk factors of breast, cervical, ovarian, and uterine cancers from 1990 to 2021. J Hematol Oncol. 2025;18:5. 39794860 10.1186/s13045-025-01660-y PMC11721161

[3] Hsu JY, Chang CJ, Cheng JS. Survival, treatment regimens and medical costs of women newly diagnosed with metastatic triple-negative breast cancer. Sci Rep. 2022;12:729. 35031634 10.1038/s41598-021-04316-2 PMC8760241

[4] Zhu S, Wu Y, Song B, Yi M, Yan Y, Mei Q, et al. Recent advances in targeted strategies for triple-negative breast cancer. J Hematol Oncol. 2023;16:100. 37641116 10.1186/s13045-023-01497-3 PMC10464091

[5] Lin NU, Claus E, Sohl J, Razzak AR, Arnaout A, Winer EP. Sites of distant recurrence and clinical outcomes in patients with metastatic triple-negative breast cancer: high incidence of central nervous system metastases. Cancer. 2008;113:2638–45. 18833576 10.1002/cncr.23930 PMC2835546

[6] Obidiro O, Battogtokh G, Akala EO. Triple negative breast cancer treatment options and limitations: future outlook. Pharmaceutics. 2023;15:1796. 37513983 10.3390/pharmaceutics15071796 PMC10384267

[7] Guo L, Kong D, Liu J, Zhan L, Luo L, Zheng W, et al. Breast cancer heterogeneity and its implication in personalized precision therapy. Exp Hematol Oncol. 2024;13:7. 38263215 10.1186/s40164-024-00472-z PMC10807152

[8] Han J, Yun J, Quan M, Kang W, Jung JG, Heo W, et al. JAK2 regulates Paclitaxel resistance in triple negative breast cancers. J Mol Med (Berl). 2021;99:1783–95. 34626199 10.1007/s00109-021-02138-3

[9] Costa RLB, Han HS, Gradishar WJ. Targeting the PI3K/AKT/mTOR pathway in triple-negative breast cancer: a review. Breast Cancer Res Treat. 2018;169:397–406. 29417298 10.1007/s10549-018-4697-y

[10] Gupta PB, Onder TT, Jiang G, Tao K, Kuperwasser C, Weinberg RA, et al. Identification of selective inhibitors of cancer stem cells by high-throughput screening. Cell. 2009;138:645–59. 19682730 10.1016/j.cell.2009.06.034 PMC4892125

[11] He L, Wick N, Germans SK, Peng Y. The role of breast cancer stem cells in chemoresistance and metastasis in triple-negative breast cancer. Cancers (Basel). 2021;13:6209. 34944829 10.3390/cancers13246209 PMC8699562

[12] Oh E, Kim YJ, An H, Sung D, Cho TM, Farrand L, et al. Flubendazole elicits anti-metastatic effects in triple-negative breast cancer via STAT3 Inhibition. Int J Cancer. 2018;143:1978–93. 29744876 10.1002/ijc.31585

[13] Ji X, Lu Y, Tian H, Meng X, Wei M, Cho WC. Chemoresistance mechanisms of breast cancer and their countermeasures. Biomed Pharmacother. 2019;114:108800. 30921705 10.1016/j.biopha.2019.108800

[14] Wang H, Guo M, Wei H, Chen Y. Targeting MCL-1 in cancer: current status and perspectives. J Hematol Oncol. 2021;14:67. 33883020 10.1186/s13045-021-01079-1 PMC8061042

[15] Balko JM, Giltnane JM, Wang K, Schwarz LJ, Young CD, Cook RS, et al. Molecular profiling of the residual disease of triple-negative breast cancers after neoadjuvant chemotherapy identifies actionable therapeutic targets. Cancer Discov. 2014;4:232–45. 24356096 10.1158/2159-8290.CD-13-0286 PMC3946308

[16] Morciano G, Giorgi C, Balestra D, Marchi S, Perrone D, Pinotti M, et al. Mcl-1 involvement in mitochondrial dynamics is associated with apoptotic cell death. Mol Biol Cell. 2016;27:20–34. 26538029 10.1091/mbc.E15-01-0028 PMC4694758

[17] Widden H, Placzek WJ. The multiple mechanisms of MCL1 in the regulation of cell fate. Commun Biol. 2021;4:1029. 34475520 10.1038/s42003-021-02564-6 PMC8413315

[18] Ding Q, He X, Xia W, Hsu JM, Chen CT, Li LY, et al. Myeloid cell leukemia-1 inversely correlates with glycogen synthase kinase-3beta activity and associates with poor prognosis in human breast cancer. Cancer Res. 2007;67:4564–71. 17495324 10.1158/0008-5472.CAN-06-1788

[19] Campbell KJ, Dhayade S, Ferrari N, Sims AH, Johnson E, Mason SM, et al. MCL-1 is a prognostic indicator and drug target in breast cancer. Cell Death Dis. 2018;9:19. 29339815 10.1038/s41419-017-0035-2 PMC5833338

[20] Winder ML, Campbell KJ. MCL-1 is a clinically targetable vulnerability in breast cancer. Cell Cycle. 2022;21:1439–55. 35349392 10.1080/15384101.2022.2054096 PMC9278428

[21] Campbell KJ, Mason SM, Winder ML, Willemsen RBE, Cloix C, Lawson H, et al. Breast cancer dependence on MCL-1 is due to its canonical anti-apoptotic function. Cell Death Differ. 2021;28:2589–600. 33785871 10.1038/s41418-021-00773-4 PMC8408186

[22] Hoy SM, Pitavastatin. A review in hypercholesterolemia. Am J Cardiovasc Drugs. 2017;17:157–68. 28130659 10.1007/s40256-017-0213-8

[23] Ramadan A, Elnour AA. Mini-review on the efficacy and safety of pitavastatin: the novel seventh statin gaining momentum. J Pharm Bioallied Sci. 2022;14:72–80. 36034492 10.4103/jpbs.jpbs_455_21 PMC9416105

[24] Kajinami K, Takekoshi N, Saito Y. Pitavastatin: efficacy and safety profiles of a novel synthetic HMG-CoA reductase inhibitor. Cardiovasc Drug Rev. 2003;21:199–215. 12931254 10.1111/j.1527-3466.2003.tb00116.x

[25] Tilija Pun N, Lee N, Song SH, Jeong CH. Pitavastatin induces cancer cell apoptosis by blocking autophagy flux. Front Pharmacol. 2022;13:854506. 35387352 10.3389/fphar.2022.854506 PMC8977529

[26] Lee N, Tilija Pun N, Jang WJ, Bae JW, Jeong CH. Pitavastatin induces apoptosis in oral squamous cell carcinoma through activation of FOXO3a. J Cell Mol Med. 2020;24:7055–66. 32406610 10.1111/jcmm.15389 PMC7299721

[27] You HY, Zhang WJ, Xie XM, Zheng ZH, Zhu HL, Jiang FZ. Pitavastatin suppressed liver cancer cells *in vitro* and *in vivo*. Onco Targets Ther. 2016;9:5383–8. 27621652 10.2147/OTT.S106906 PMC5010166

[28] Haciseyitoglu AO, Dogan TC, Dilsiz SA, Canpinar H, Eken A, Bucurgat UU. Pitavastatin induces caspase-mediated apoptotic death through oxidative stress and DNA damage in combined with cisplatin in human cervical cancer cell line. J Appl Toxicol. 2024;44:623–40. 38053498 10.1002/jat.4565

[29] Brinkmann K, Grabow S, Hyland CD, Teh CE, Alexander WS, Herold MJ, et al. The combination of reduced MCL-1 and standard chemotherapeutics is tolerable in mice. Cell Death Differ. 2017;24:2032–43. 28800129 10.1038/cdd.2017.125 PMC5686343

[30] Sancho M, Leiva D, Lucendo E, Orzaez M. Understanding MCL1: from cellular function and regulation to pharmacological inhibition. FEBS J. 2022;289:6209–34. 34310025 10.1111/febs.16136 PMC9787394

[31] Xiao Y, Nimmer P, Sheppard GS, Bruncko M, Hessler P, Lu X, et al. MCL-1 is a key determinant of breast cancer cell survival: validation of MCL-1 dependency utilizing a highly selective small molecule inhibitor. Mol Cancer Ther. 2015;14:1837–47. 26013319 10.1158/1535-7163.MCT-14-0928

[32] Saito Y. Pitavastatin: an overview. Atheroscler Suppl. 2011;12:271–6. 22152281 10.1016/S1567-5688(11)70886-8

[33] Mallick DJ, Eastman A, Cancers. (Basel). 2020;12:2298. 10.3390/cancers12082298 PMC7464284 32824203

[34] Yi X, Sarkar A, Kismali G, Aslan B, Ayres M, Iles LR, et al. AMG-176, an Mcl-1 Antagonist, shows preclinical efficacy in chronic lymphocytic leukemia. Clin Cancer Res. 2020;26:3856–67. 31937611 10.1158/1078-0432.CCR-19-1397 PMC7358119

[35] Tantawy SI, Timofeeva N, Sarkar A, Gandhi V. Targeting MCL-1 protein to treat cancer: opportunities and challenges. Front Oncol. 2023;13:1226289. 37601693 10.3389/fonc.2023.1226289 PMC10436212

[36] Ginestier C, Hur MH, Charafe-Jauffret E, Monville F, Dutcher J, Brown M, et al. ALDH1 is a marker of normal and malignant human mammary stem cells and a predictor of poor clinical outcome. Cell Stem Cell. 2007;1:555–67. 18371393 10.1016/j.stem.2007.08.014 PMC2423808

[37] Li W, Ma H, Zhang J, Zhu L, Wang C, Yang Y. Unraveling the roles of CD44/CD24 and ALDH1 as cancer stem cell markers in tumorigenesis and metastasis. Sci Rep. 2017;7:13856. 29062075 10.1038/s41598-017-14364-2 PMC5653849

[38] Quintero-Fabian S, Arreola R, Becerril-Villanueva E, Torres-Romero JC, Arana-Argaez V, Lara-Riegos J, et al. Role of matrix metalloproteinases in angiogenesis and cancer. Front Oncol. 2019;9:1370. 31921634 10.3389/fonc.2019.01370 PMC6915110

[39] Kwon MJ. Matrix metalloproteinases as therapeutic targets in breast cancer. Front Oncol. 2022;12:1108695. 36741729 10.3389/fonc.2022.1108695 PMC9897057

[40] Kim S, Park JM, Park S, Jung E, Ko D, Park M, et al. Suppression of TNBC metastasis by doxazosin, a novel dual inhibitor of c-MET/EGFR. J Exp Clin Cancer Res. 2023;42:292. 37924112 10.1186/s13046-023-02866-z PMC10625208

[41] Pushpakom S, Iorio F, Eyers PA, Escott KJ, Hopper S, Wells A, et al. Drug repurposing: progress, challenges and recommendations. Nat Rev Drug Discov. 2019;18:41–58. 30310233 10.1038/nrd.2018.168

[42] Xia Y, Sun M, Huang H, Jin WL. Drug repurposing for cancer therapy. Signal Transduct Target Ther. 2024;9:92. 38637540 10.1038/s41392-024-01808-1 PMC11026526

[43] Chen LW, Lin CS, Tsai MC, Shih SF, Lim ZW, Chen SJ, et al. Pitavastatin exerts potent Anti-Inflammatory and Immunomodulatory effects via the suppression of AP-1 signal transduction in human T cells. Int J Mol Sci. 2019;20:3534. 31330988 10.3390/ijms20143534 PMC6678418

[44] Cheng BF, Gao YX, Lian JJ, Guo DD, Liu TT, Xie YF, et al. Anti-inflammatory effects of Pitavastatin in interleukin-1beta-induced SW982 human synovial cells. Int Immunopharmacol. 2017;50:224–9. 28692879 10.1016/j.intimp.2017.06.032

[45] Inamoto S, Yoshioka T, Yamashita C, Miyamura M, Mori T, Ukimura A, et al. Pitavastatin reduces oxidative stress and attenuates intermittent hypoxia-induced left ventricular remodeling in lean mice. Hypertens Res. 2010;33:579–86. 20300107 10.1038/hr.2010.36

[46] Fatehi Hassanabad A. Current perspectives on statins as potential anti-cancer therapeutics: clinical outcomes and underlying molecular mechanisms. Transl Lung Cancer Res. 2019;8:692–9. 31737505 10.21037/tlcr.2019.09.08 PMC6835101

[47] Catapano AL. Pitavastatin - pharmacological profile from early phase studies. Atheroscler Suppl. 2010;11:3–7. 21193152 10.1016/S1567-5688(10)71063-1

[48] Pitavastatin. Uses, Interactions, Mechanism of Action, DrugBank. https://go.drugbank.com/drugs/DB08860

[49] FDA Approved Drug Products. NIKITATM (pitavastatin) tablets, for oral use. https://www.accessdata.fda.gov/drugsatfda_docs/label/2023/209875s003lbl.pdf

[50] Szlavik Z, Csekei M, Paczal A, Szabo ZB, Sipos S, Radics G, et al. Discovery of S64315, a potent and selective Mcl-1 inhibitor. J Med Chem. 2020;63:13762–95. 33146521 10.1021/acs.jmedchem.0c01234

[51] Willis SN, Chen L, Dewson G, Wei A, Naik E, Fletcher JI, et al. Proapoptotic bak is sequestered by Mcl-1 and Bcl-xL, but not Bcl-2, until displaced by BH3-only proteins. Genes Dev. 2005;19:1294–305. 15901672 10.1101/gad.1304105 PMC1142553

[52] Gelinas C, White E. BH3-only proteins in control: specificity regulates MCL-1 and BAK-mediated apoptosis. Genes Dev. 2005;19:1263–8. 15937216 10.1101/gad.1326205

[53] Lee KM, Giltnane JM, Balko JM, Schwarz LJ, Guerrero-Zotano AL, Hutchinson KE et al. MYC and MCL1 cooperatively promote chemotherapy-resistant breast cancer stem cells via regulation of mitochondrial oxidative phosphorylation. Cell Metab. 2017;26:633–47.e7. 10.1016/j.cmet.2017.09.009 PMC5650077 28978427

[54] Opferman JT, Iwasaki H, Ong CC, Suh H, Mizuno S, Akashi K, et al. Obligate role of anti-apoptotic MCL-1 in the survival of hematopoietic stem cells. Science. 2005;307:1101–4. 15718471 10.1126/science.1106114

[55] Rasmussen ML, Kline LA, Park KP, Ortolano NA, Romero-Morales AI, Anthony CC, et al. A Non-apoptotic function of MCL-1 in promoting pluripotency and modulating mitochondrial dynamics in stem cells. Stem Cell Rep. 2018;10:684–92. 10.1016/j.stemcr.2018.01.005 PMC5918190 29429957

[56] Zhang H, Li G, Chen G, Zhang Y, Pan J, Tang H, et al. Targeting Mcl-1 inhibits survival and self-renewal of hepatocellular cancer stem-like cells. Clin Res Hepatol Gastroenterol. 2019;43:292–300. 30528319 10.1016/j.clinre.2018.11.004

[57] Bolomsky A, Vogler M, Kose MC, Heckman CA, Ehx G, Ludwig H, et al. MCL-1 inhibitors, fast-lane development of a new class of anti-cancer agents. J Hematol Oncol. 2020;13:173. 33308268 10.1186/s13045-020-01007-9 PMC7731749

[58] Liu S, Cong Y, Wang D, Sun Y, Deng L, Liu Y, et al. Breast cancer stem cells transition between epithelial and mesenchymal States reflective of their normal counterparts. Stem Cell Rep. 2014;2:78–91. 10.1016/j.stemcr.2013.11.009 PMC3916760 24511467

[59] Mani SA, Guo W, Liao MJ, Eaton EN, Ayyanan A, Zhou AY, et al. The epithelial-mesenchymal transition generates cells with properties of stem cells. Cell. 2008;133:704–15. 18485877 10.1016/j.cell.2008.03.027 PMC2728032

[60] Scheel C, Weinberg RA. Cancer stem cells and epithelial-mesenchymal transition: concepts and molecular links. Semin Cancer Biol. 2012;22:396–403. 22554795 10.1016/j.semcancer.2012.04.001 PMC6220425

[61] Jin W. Role of JAK/STAT3 signaling in the regulation of metastasis, the transition of cancer stem cells, and chemoresistance of cancer by epithelial-mesenchymal transition. Cells. 2020;9:217. 31952344 10.3390/cells9010217 PMC7017057

[62] Roninson IB. The role of the MDR1 (P-glycoprotein) gene in multidrug resistance *in vitro* and *in vivo*. Biochem Pharmacol. 1992;43:95–102. 1346497 10.1016/0006-2952(92)90666-7

[63] Vaidyanathan A, Sawers L, Gannon AL, Chakravarty P, Scott AL, Bray SE, et al. ABCB1 (MDR1) induction defines a common resistance mechanism in paclitaxel- and olaparib-resistant ovarian cancer cells. Br J Cancer. 2016;115:431–41. 27415012 10.1038/bjc.2016.203 PMC4985349

[64] Xu F, Wang F, Yang T, Sheng Y, Zhong T, Chen Y. Differential drug resistance acquisition to doxorubicin and paclitaxel in breast cancer cells. Cancer Cell Int. 2014;14:142. 25550688 10.1186/s12935-014-0142-4 PMC4279688

[65] Wang WJ, Li CF, Chu YY, Wang YH, Hour TC, Yen CJ, et al. Inhibition of the EGFR/STAT3/CEBPD axis reverses cisplatin Cross-resistance with Paclitaxel in the urothelial carcinoma of the urinary bladder. Clin Cancer Res. 2017;23:503–13. 27435393 10.1158/1078-0432.CCR-15-1169

[66] Ji L, Liu X, Zhang S, Tang S, Yang S, Li S, et al. The novel Triazolonaphthalimide derivative LSS-11 synergizes the anti-proliferative effect of Paclitaxel via STAT3-dependent MDR1 and MRP1 downregulation in chemoresistant lung cancer cells. Molecules. 2017;22:1822. 29072615 10.3390/molecules22111822 PMC6150343

[67] Zhang X, Xiao W, Wang L, Tian Z, Zhang J. Deactivation of signal transducer and activator of transcription 3 reverses chemotherapeutics resistance of leukemia cells via down-regulating P-gp. PLoS ONE. 2011;6:e20965. 21677772 10.1371/journal.pone.0020965 PMC3108986

[68] Fang Z, Chen W, Yuan Z, Liu X, Jiang H. LncRNA-MALAT1 contributes to the cisplatin-resistance of lung cancer by upregulating MRP1 and MDR1 via STAT3 activation. Biomed Pharmacother. 2018;101:536–42. 29505924 10.1016/j.biopha.2018.02.130

[69] Becker TM, Boyd SC, Mijatov B, Gowrishankar K, Snoyman S, Pupo GM, et al. Mutant B-RAF-Mcl-1 survival signaling depends on the STAT3 transcription factor. Oncogene. 2014;33:1158–66. 23455323 10.1038/onc.2013.45

