# Supplementary material for: Pitavastatin is a novel Mcl-1 inhibitor that overcomes paclitaxel resistance in triple-negative breast cancer
Source: Exp Hematol Oncol. 2025 Oct 22;14:125. doi: 10.1186/s40164-025-00716-6 (PMC12548168; doi:10.1186/s40164-025-00716-6)
Supplement: Supplementary file 2 — Supplementary Material 2 [file 40164_2025_716_MOESM2_ESM.docx]

**Supplementary information 1**

**Pitavastatin is a Novel Mcl-1 Inhibitor that Overcomes Paclitaxel Resistance in Triple-negative Breast Cancer**

Dongmi Ko^1,2^, Soeun Park^1,2^, Minsu Park^1,2^, Seongjae Kim^1,2^, Jung Min Park^1,2^, Juyeon Seo^1,2^, Kee Dal Nam^1,3^, Yong Koo Kang^1,3^, Lee Farrand^4^, Eunsun Jung^1,2*^, Yoon-Jae Kim^1,2,3*^, Ji Young Kim^1,3*^, and Jae Hong Seo^1,2,3*^

**Supplementary Figures and Legends**

***Supplementary Fig. S1***

**
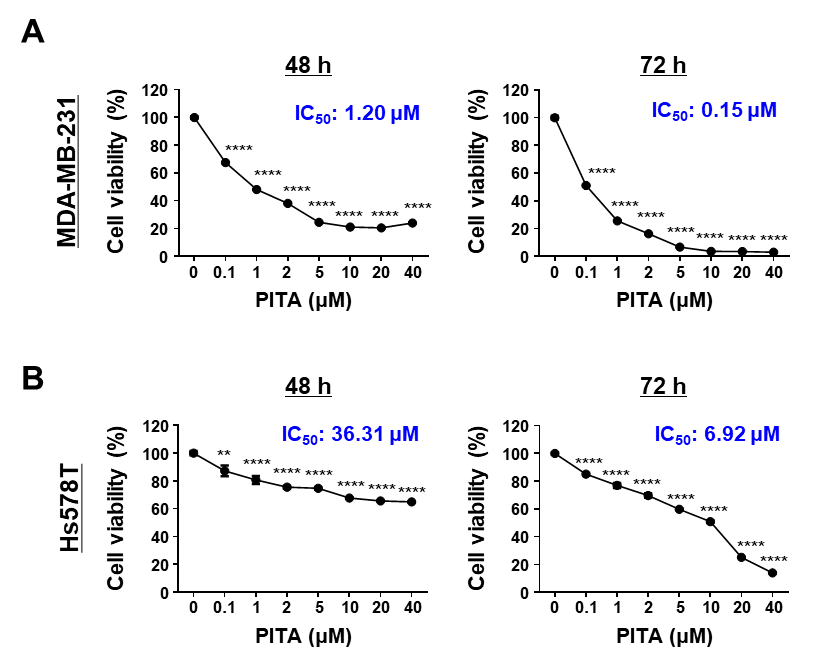
**

**Supplementary Fig. S1: PITA reduces viability in additional human TNBC cell lines.**

**A-B** MDA-MB-231 (**A**) and Hs578T (**B**) cells treated with PITA (0–40 μM) for 48 or 72 h. Cell viability and IC_50_ values were determined by MTS assay. Data represent mean ± SEM from three independent experiments and were analyzed by one-way ANOVA with Bonferroni’s post hoc test (***p* < 0.01).

***Supplementary Fig. S2***

**
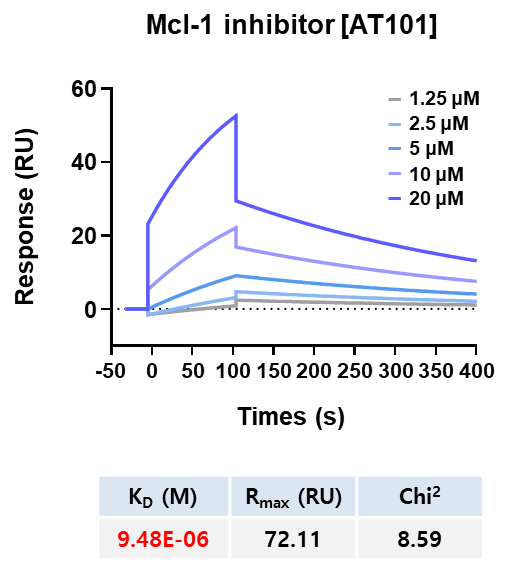
**

**Supplementary Fig. S2: Surface plasmon resonance (SPR) binding curves for the interaction between AT101 and Mcl-1.**

The indicated concentrations of Mcl-1 inhibitor AT101 (1.25-20 µM) were passed over immobilized human Mcl-1 protein on HC1000M sensor chips. Kinetic interactions of AT101 (K_D_ = 9.48 µM) with Mcl-1 were determined by SPR analysis.

***Supplementary Fig. S3***

***
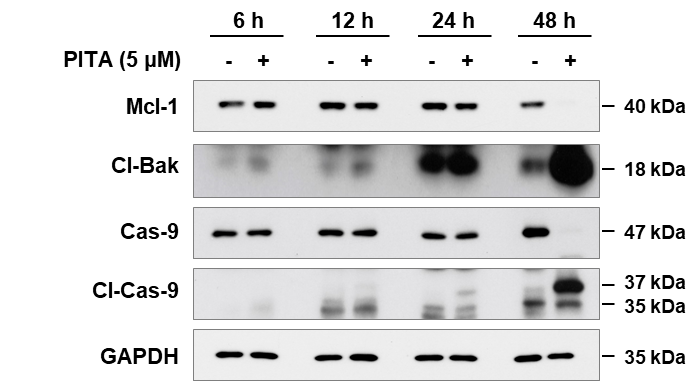
***

**Supplementary Fig. S3: Changes in the expression of Mcl-1, cleaved Bak, caspase-3 and cleaved caspase-3 protein in a time-dependent manner.**

BT549 cells were treated with PITA (5 μM) for the indicated times (6, 12, 24, and 48 h), and protein expression levels were determined by immunoblot analysis. Cl-Bak, cleaved Bak; Cas-9, caspase-9; Cl-Cas-9, cleaved caspase-9.

***Supplementary Fig. S4***

***
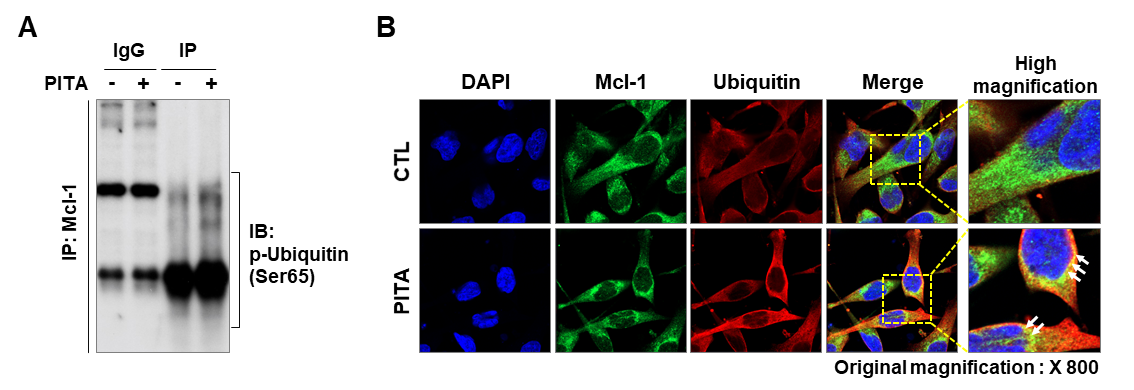
***

**Supplementary Fig. S4: Ubiquitin-mediated degradation of Mcl-1 by PITA.**

**A** BT549 cells were treated with PITA (5 μM) for 24 h. Cell lysates were immunoprecipitated (IP) with an anti-Mcl-1 antibody and analyzed by immunoblotting (IB) with a phospho-ubiquitin (Ser65) antibody. IgG, normal mouse immunoglobulin G. **B** BT549 cells were co-immunostained for Mcl-1 (green) and ubiquitin (red) with DAPI (blue) following exposure to PITA (5 μM) for 24 h. Co-localization of Mcl-1 and ubiquitin in the plasma membrane is seen as yellow signals (white arrows) at high magnification (× 2000).

***Supplementary Fig. S5***

***
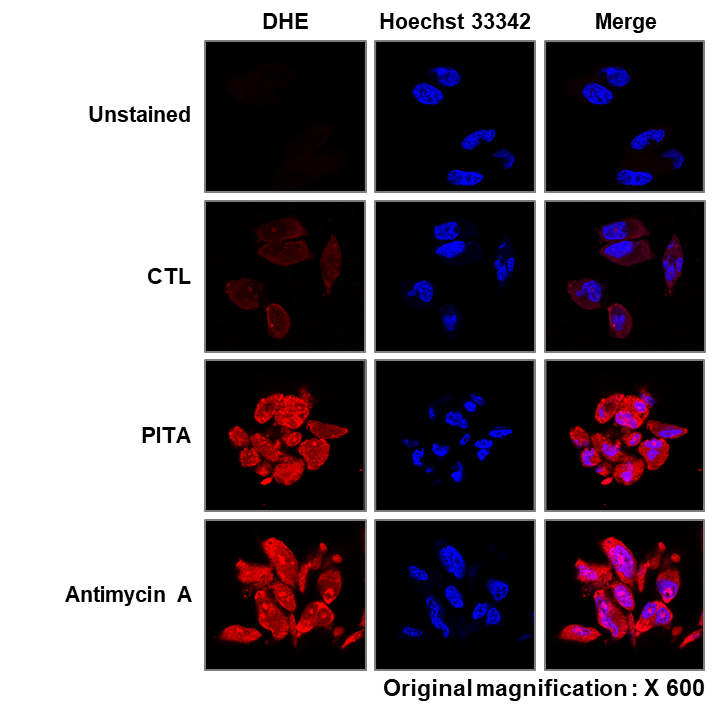
***

**Supplementary Fig. S5: Immunofluorescence analysis for mitochondrial ROS accumulation in BT549 cells following exposure to PITA.**

BT549 cells were treated with PITA (5 µM), antimycin A (10 µM), or control vehicle (DMSO) for 3 h and immunostained for dihydroethidium (DHE, red) with Hoechst 33342 (nuclei, blue).

***Supplementary Fig. S6***

**
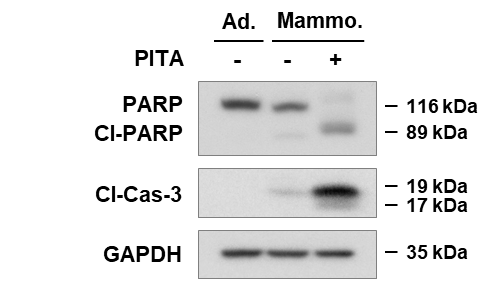
**

**Supplementary Fig. S6: Changes in the expression of apoptosis-related proteins in 4T1-mammospheres.**

Immunoblot analyses for PARP, cleaved-PARP, cleaved-caspase-3 expression in 4T1-mammospheres following exposure to PITA (10 μM, 3 days).

***Supplementary Fig. S7***

*
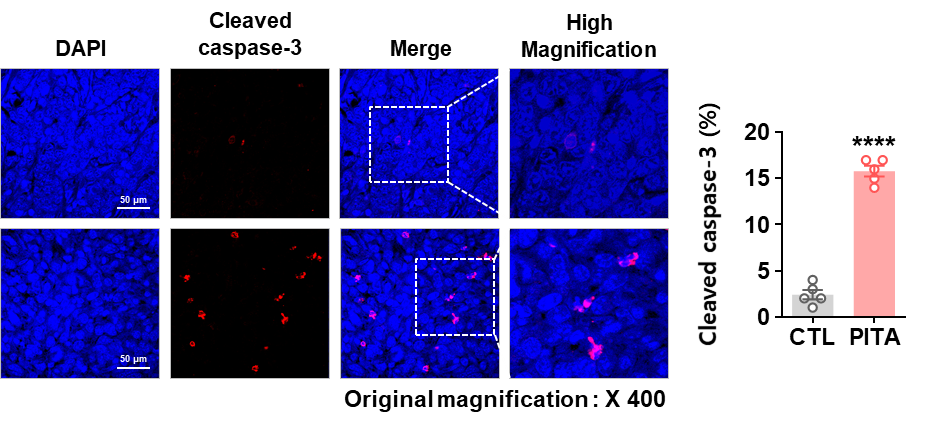
*

**Supplementary Fig. S7: Influence of PITA on active caspase-3 expression in allograft tumors derived from 4T1 mammospheres.**

Immunohistochemical analysis for cleaved caspase-3 in allograft tumors. Tissue sections were immunostained for cleaved caspase-3 (red) with DAPI (blue) (*****p* < 0.0001).

***Supplementary Fig. S8***

**
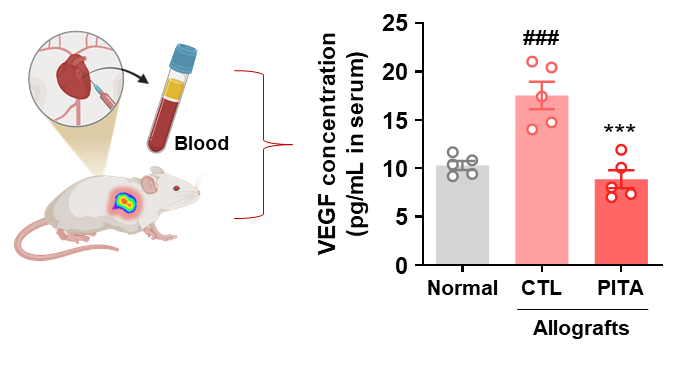
**

**Supplementary Fig. S8: Effect of PITA on VEGF levels in 4T1 allografts.**

Changes in serum levels of VEGF in tumor-bearing mice following PITA administration. Normal mouse serum was used as a negative control (normal mice vs. control; ###*p* < 0.001, control vs. PITA-treated group; ****p* < 0.001).

***Supplementary Fig. S9***

*
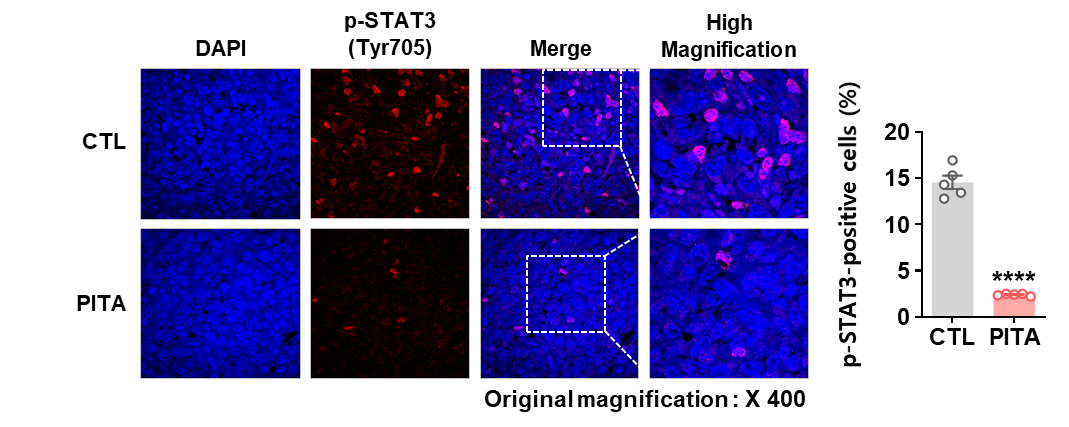
*

**Supplementary Fig. S9: Influence of PITA on phospho-STAT3 expression in allograft tumors derived from 4T1 mammospheres.**

Immunohistochemical analysis for phospho-STAT3 (Tyr705) in allograft tumors. Tissue sections were immunostained for phospho-STAT3 (red) with DAPI (blue), and p-STAT3-positive cells were counted (*****p* < 0.0001).

***Supplementary Fig. S10***

**
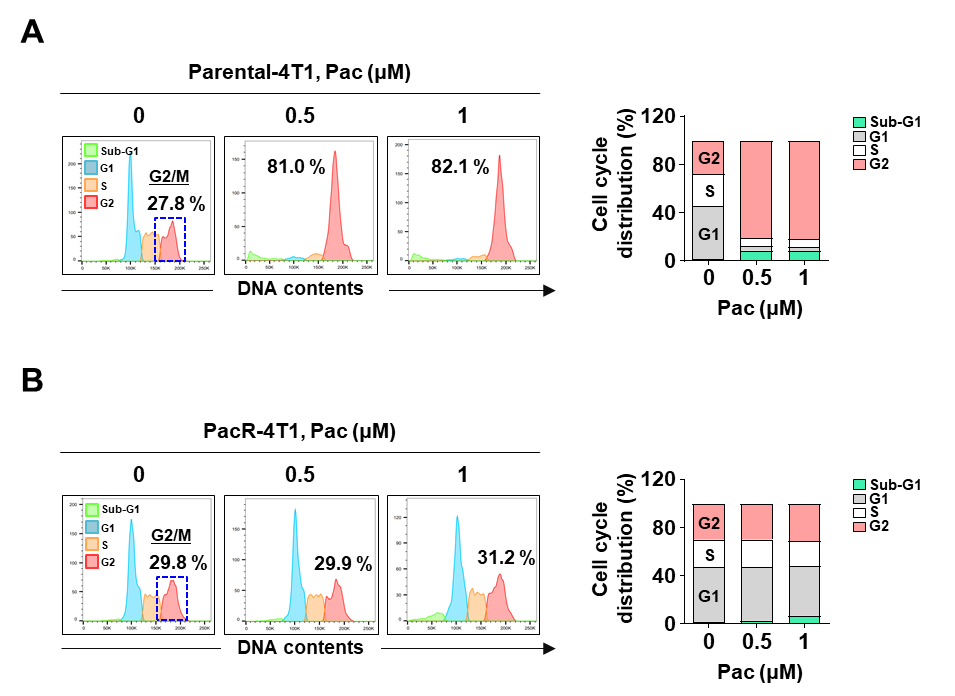
**

**Supplementary Fig. S10: Comparison of cell cycle distribution in parental-4T1 and PacR-4T1 cells after treatment with Pac.**

Parental- (**A**) and PacR-4T1 (**B**) cells were treated with Pac (0-1 μM) for 12 h, and cell cycle distributions were determined by PI staining using flow cytometry. Representative histogram images show the proportion of cells in each phase of the cell cycle (G2/M fractions, blue square). The bar graphs in the right panels represent cell cycle distribution.

***Supplementary Fig. S11***

***
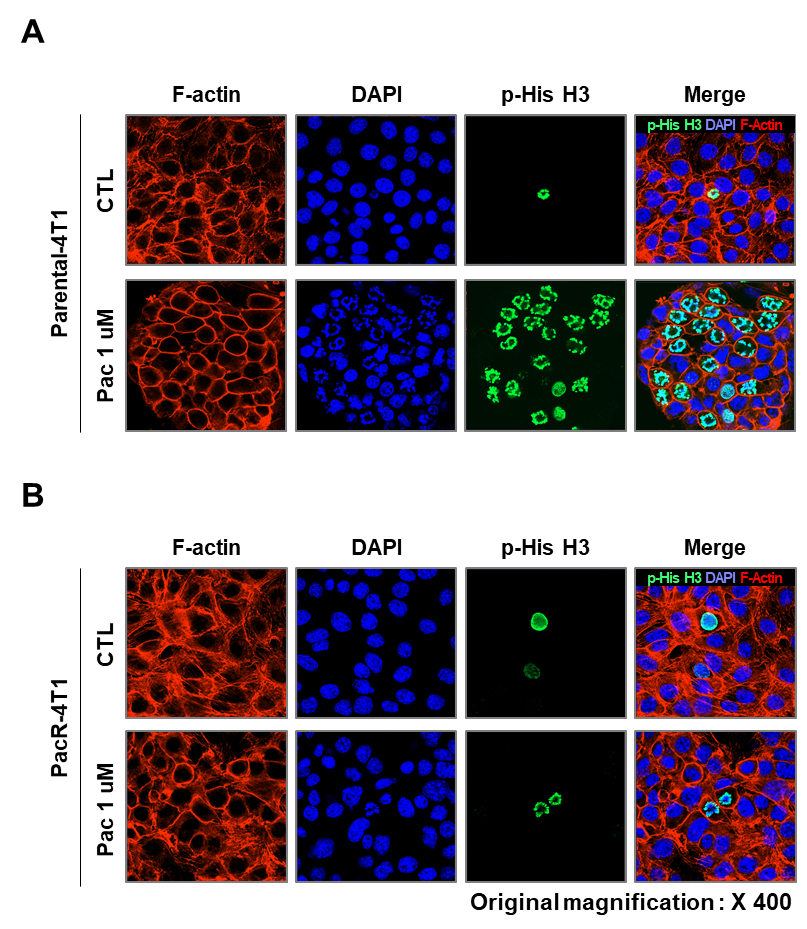
***

**Supplementary Fig. S11: Immunocytochemical analysis for p-His H3 (green) in parental- and PacR-4T1 cells following exposure to Pac.**

Parental- (**A**) and PacR-4T1 (**B**) cells were treated with Pac (1 μM) for 12 h, and immunostained for p-His H3 (green), F-actin (red), and DAPI (blue).

***Supplementary Fig. S12***

**
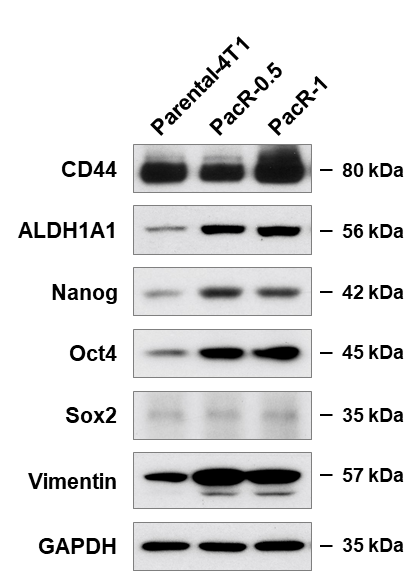
**

**Supplementary Fig. S12: Changes in the expression of CSC-related proteins in PacR-4T1 cells.**

Immunoblot analyses for CD44, ALDH1A1, Nanog, Oct4, Sox2, and vimentin protein expression in parental- and PacR-4T1 cells (PacR-0.5, resistant to 0.5 μM of Pac; PacR-1, resistant to 1 μM of Pac).

***Supplementary Fig. S13***

**
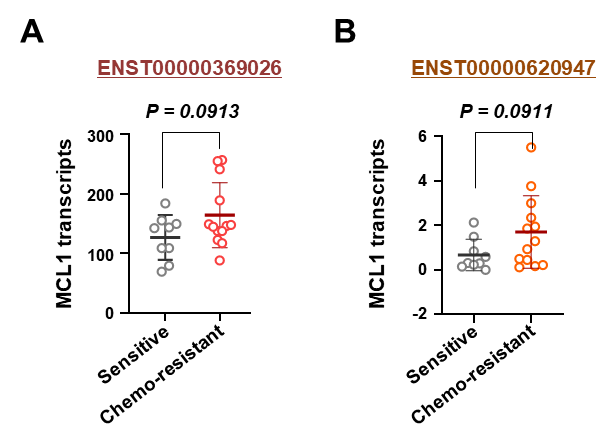
**

**Supplementary Fig. S13: Differences in MCL1 transcript expression between taxane-sensitive and -resistant breast cancer patients.**

**A-B** Analysis of MCL1 transcript expression using the publicly available GEO dataset GSE162187. Expression levels of MCL1 isoform ENST00000369026 (**A**) and ENST00000620947 (**B**) in taxane-sensitive and -resistant patients are presented as log₂-transformed TPM values. Statistical significance was assessed using an unpaired two-tailed t-test. [gray dots, taxane-sensitive breast cancer patients; red/orange dots, taxane-resistant breast cancer patients].

***Supplementary Fig. S14***

**
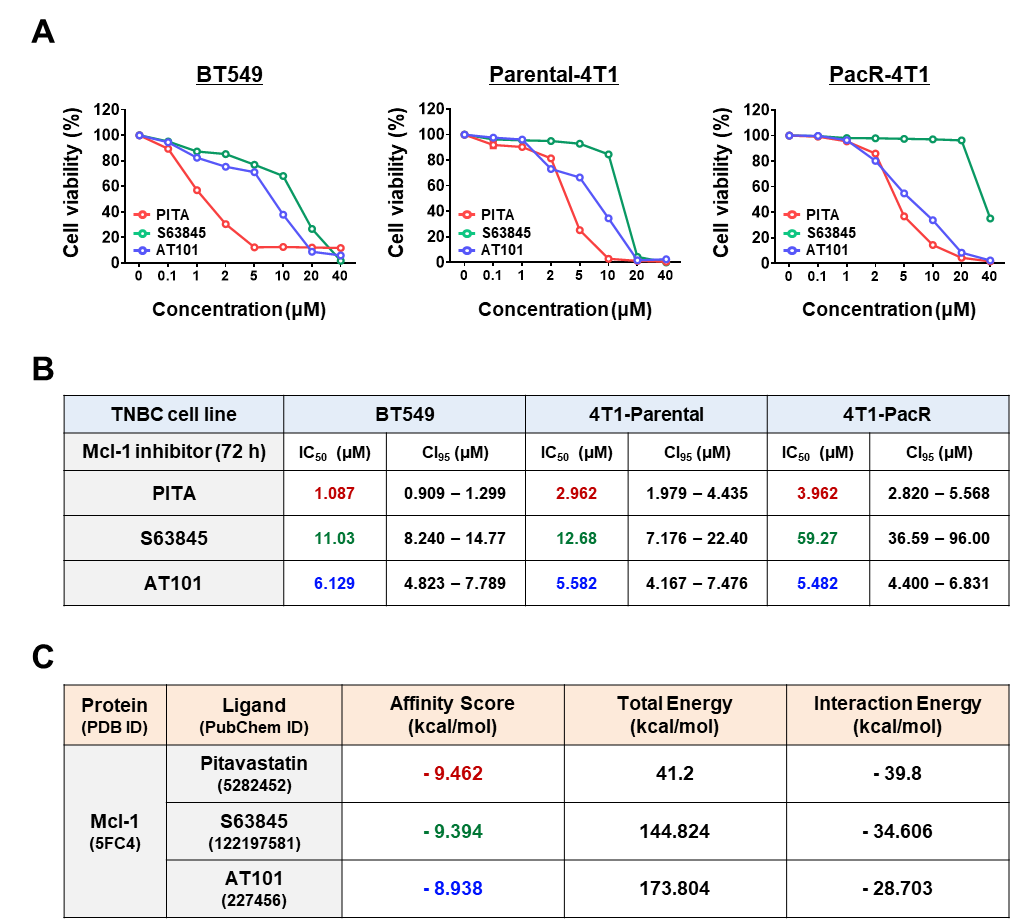
**

**Supplementary Fig. S14: Comparison of cell viability in paclitaxel-sensitive and -resistant TNBC cells following treatment with PITA and Mcl-1 inhibitors.**

**A-C** BT549, Parental-4T1 and PacR-4T1 cells were treated with PITA, S63845 or AT101 (0–40 μM, 72 h). **A** Cell viability was determined by MTS assay. **B** IC_50_ values for cell viability. **C** *In silico* molecular docking simulation of PITA, S63845, and AT101 with Mcl-1 (PDB: 5FC4).

***Supplementary Fig. S15***

**
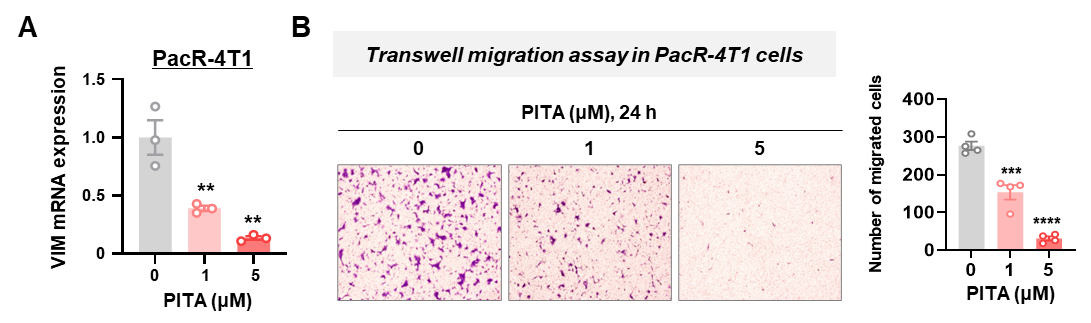
**

**Supplementary Fig. S15: PITA downregulates vimentin (VIM) transcripts and suppresses migration in PacR-4T1 cells.**

**A** mRNA expression levels of vimentin (VIM) in PacR-4T1 cells following treatment with PITA (0-5 μM, ***p* < 0.01). **B** Transwell migration assay of PacR-4T1 cells treated with PITA (0-5 μM). Migrated cells were stained with crystal violet and quantified (****p* < 0.001).

***Supplementary Fig. S16***

**
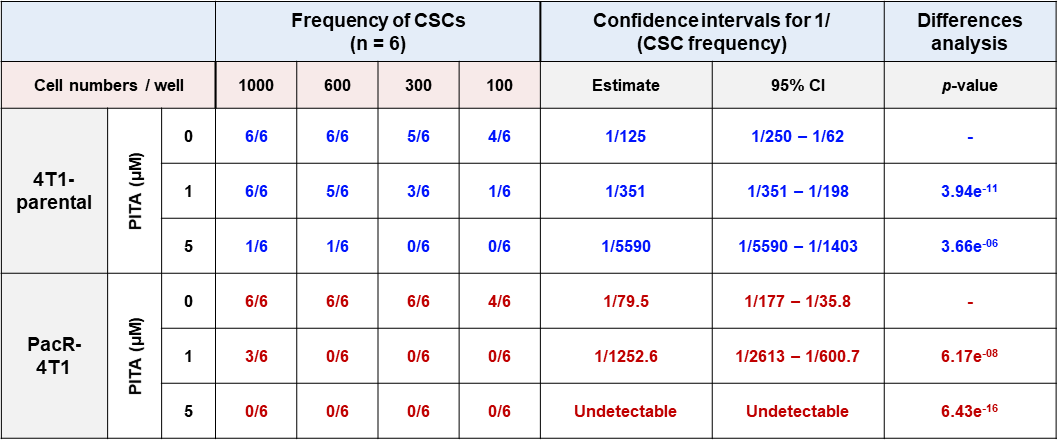
**

**Supplementary Fig. S16: Extreme limiting dilution analysis (ELDA) of CSC frequency in 4T1-parental and PacR-4T1 cells.**

Cells were plated at different densities (1000, 600, 300, or 100 cells per well; n = 6 wells per dilution), and the frequency of wells containing CSC-derived mammospheres was determined. CSC frequency and the estimated number of cells required to generate one CSC-derived sphere were calculated using ELDA software, with 95% confidence intervals shown. Statistical differences between groups were assessed within the ELDA framework, and p-values are indicated.

***Supplementary Fig. S17***

**
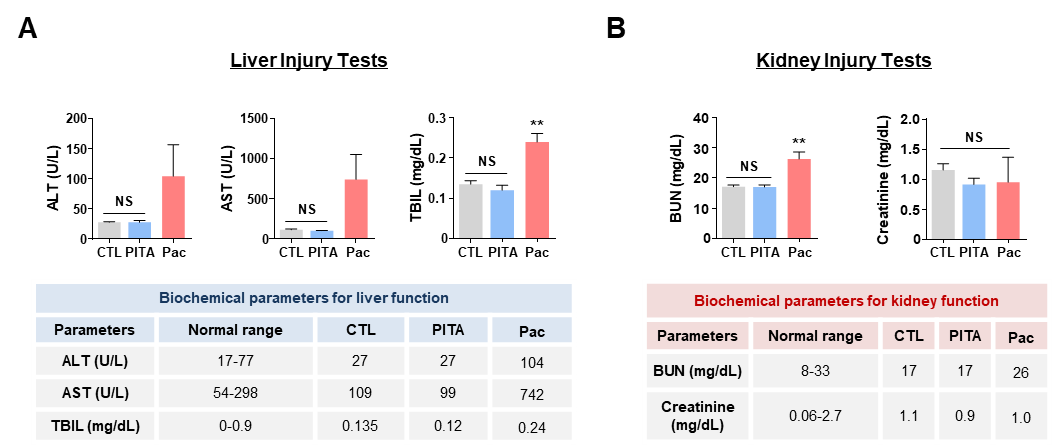
**

**Supplementary Fig. S17: Effects of PITA or Pac on serum biochemical parameters of liver and kidney function.**

**A-B** Comparison of serum biochemical parameters of liver (**A**) and kidney injury (**B**) in control, PITA- or Pac-treated mice. Hepatorenal toxicity was determined via serum levels of ALT, AST, total bilirubin (TBIL), BUN, and creatinine (***p* < 0.01). The results are presented as mean ± SEM of at least three independent experiments and analyzed by one-way ANOVA followed by Bonferroni's *post hoc* test.

***Supplementary Fig. S18***

**
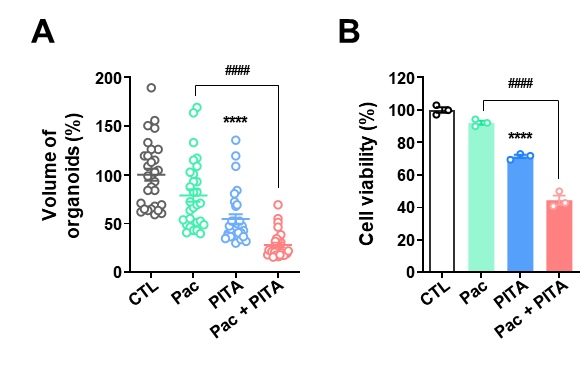
**

**Supplementary Fig. S18: Effect of PITA and/or paclitaxel on organoid growth and viability.**

**A** Quantification of organoid volume after 7 days of treatment with PITA and/or paclitaxel (*****p* < 0.0001; PITA only vs control; NS, Pac only vs control; ####*p* < 0.0001, Pac only vs combination). **B** Organoid cell viability evaluated using CellTiter-Glo 3D after 7-days of treatment with PITA and/or paclitaxel (*****p* < 0.0001; PITA only vs control; NS, Pac only vs control; ####*p* < 0.001, Pac only vs combination).
