# Supplementary material for: Pitavastatin is a novel Mcl-1 inhibitor that overcomes paclitaxel resistance in triple-negative breast cancer
Source: Exp Hematol Oncol. 2025 Oct 22;14:125. doi: 10.1186/s40164-025-00716-6 (PMC12548168; doi:10.1186/s40164-025-00716-6)
Supplement: Supplementary file 3 — Supplementary Material 3 [file 40164_2025_716_MOESM3_ESM.docx]

**Supplementary Information 2**

**Pitavastatin is a Novel Mcl-1 Inhibitor that Overcomes Paclitaxel Resistance in Triple-negative Breast Cancer**

Dongmi Ko^1,2^, Soeun Park^1,2^, Minsu Park^1,2^, Seongjae Kim^1,2^, Jung Min Park^1,2^, Juyeon Seo^1,2^, Kee Dal Nam^1,3^, Yong Koo Kang^1,3^, Lee Farrand^4^, Eunsun Jung^1,2*^, Yoon-Jae Kim^1,2,3*^, Ji Young Kim^1,3*^, and Jae Hong Seo^1,2,3*^

**Supplementary Methods**

***Reagents, materials, and antibodies***

Pitavastatin (PITA) was obtained from Cayman Chemical Company (East Ellsworth Road Ann Arbor, MI). Paclitaxel, AT-101 and S63845 was purchased from Selleckchem (Radnor, PA). Triton X-100, Tween-20, propidium iodide (PI), phosphate-buffered saline (PBS) tablets, corn oil, and dimethyl sulfoxide (DMSO) were obtained from Sigma-Aldrich (St. Louis, MO). RNase A was purchased from Invitrogen (Carlsbad, CA). Phosphatase inhibitor and protease inhibitor cocktail tablets were obtained from Roche Applied Sciences (Penzberg, GER). Primary antibodies targeted the following proteins: ALDH1A1, CD31, CD44, CD49f, STAT3, p-STAT3, Ki-67, vimentin, Bcl-2, ubiquitin and P-gp (Abcam, MA); AKT, phospho-AKT (S473), JAK2, phospho-histone H3 (Ser10), PARP, cleaved-PARP, cleaved-caspase-3, cleaved-caspase-7, cleaved-caspase-8, caspase-9, cleaved-caspase-9, Mcl-1, phospho-ubiquitin (Ser65), Bak, Nanog, Oct4 and Sox2 (Cell Signaling Technology, MA); ALDH1A1, Mcl-1, cyclin D1, survivin, cytochrome c and Tom 20 (Santa Cruz Biotechnology, CA); Texas Red-X Phalloidin (F-actin) and GAPDH (Invitrogen). Secondary antibodies were horseradish peroxidase (HRP)-conjugated anti-rabbit and mouse IgG (Bio-Rad Laboratories, CA); and Alexa Fluor-488 and -594 goat anti-mouse IgG (Invitrogen).

***Breast cancer cell culture***

The human TNBC cell line MDA-MB-231 (PerkinElmer Inc. USA), Hs578T (American Type Culture Collection, ATCC), BT549 and murine mammary carcinoma 4T1-Luc (Japanese Collection of Research Bioresources Cell Bank, Japan) were cultured in MEM or RPMI 1640 (Gibco, MD) supplemented with 10% fetal bovine serum (FBS) and streptomycin-penicillin (100 U/mL) at 37°C with 5% CO_2_. Emergent resistance to paclitaxel in the 4T1 cell line, named PacR-4T1, was established through continuous induction with stepwise escalating concentrations of paclitaxel, ranging from 140 to 1000 nM over a period of 6 months. All cell lines were passaged for less than 6 months after resuscitation and were used from passages 3 to 20. All cell lines were authenticated by short tandem repeat (STR) profiling by Macrogen Inc. (Seoul, South Korea).

***Cell viability assay***

Cell viability was measured using the CellTiter 96® Aqueous One Solution Cell Proliferation Assay [MTS, 3-(4, 5-dimethylthiazol-2-yl)-5-(3-carboxymethoxyphenyl)-2-(4-sulfophenyl)-2H-tetrazolium] (Promega, WI) according to the manufacturer’s instructions. The quantity of formazan product was determined by measuring the absorbance at 490 nm with a microplate reader (Agilent BioTek 800 TS, Agilent Technologies, VT).

***Sub-G1 analysis and Annexin V/PI assay***

Cells were harvested and fixed with 95% ethanol containing 0.5% Tween-20 for 24 h, and incubated with propidium iodide (PI, 50 mg/mL) and RNase (50 mg/mL) for 30 min. The annexin V/PI assay was assessed using a FITC-conjugated Annexin V apoptosis detection kit (BD Biosciences, NJ) in accordance with the manufacturer’s protocol. Stained cells were analyzed by flow cytometry using a BD LSRFortessa^TM^ X-20 Cell Analyzer (BD Biosciences).

***Aldefluor-positivity assay,*** ***CD44^high^/CD24^low^ and CD49f^high^/CD24^high^ staining***

ALDH1 activity was analyzed with an Aldefluor assay kit (Stem Cell Technologies, Canada) according to the manufacturer’s instructions. Cells were incubated for 45 min at 37℃ in Aldefluor assay buffer containing ALDH1 protein substrate BODIPY-aminoacetaldehyde (BAAA, 1 µM / 0.5×10^6^ cells). The ALDH1-specific inhibitor, diethylamino-benzaldehyde (DEAB; 50 mM), was defined as the baseline of Aldefluor fluorescence with flow cytometry. To determine the CD44^high^/CD24^low^ and CD49f^high^/CD24^high^ phenotypes, cells were immunostained with FITC- and PE-conjugated anti-mouse IgG or FITC-conjugated anti-CD24 and PE-conjugated anti-CD44 or CD49f antibodies (BD Biosciences) for 30 min at 4°C and analyzed by flow cytometry.

***RNA extraction and RT-qPCR***

Total RNA was isolated from parental-4T1 and PacR-4T1 cells treated with PITA (0-5 μM) for 48 h using NecleoZol reagent (Macherey-Nagel) in accordance with the manufacturer’s protocol. Complementary DNA (cDNA) was synthesized from 1 μg of RNA using the Hisense™ cDNA Synthesis Master Mix (CellSafe, Korea). Quantitative PCR was conducted for VIM, TWIST1, SNAI1 and the reference gene GAPDH using SYBR-Green PCR Master Mix (Applied Biosystems) on a Quant6 Real-Time PCR system (Applied Biosystems). The amplification protocol consisted of an initial denaturation at 95°C for 3 min, followed by 40 cycles of 95°C for 10 sec and 58°C for 1 min. Each reaction was performed in triplicate. The PCR primers used were as follows: VIM (forward, cgtccacacgcacctacag and reverse, gggggatgaggaatagaggct), GAPDH (forward, aggtcggtgtgaacggatttg and reverse, tgtagaccatgtagttgaggtca)

***Immunoblot analysis***

Cells were solubilized in lysis buffer [30 mM NaCl, 0.5% Triton X-100, 50 mM Tris-HCl (pH 7.4)] containing a phosphatase and protease inhibitor cocktail for 45 min on ice. After centrifugation at 15,000 rpm and 4°C for 20 min, the supernatant was collected, and protein concentrations were measured using a Bradford protein assay kit (Bio-Rad Laboratories). Equal quantities of protein (25 μg) were subjected to SDS-PAGE and electrotransferred onto a polyvinylidene fluoride (PVDF) membrane (Millipore, MA). The membranes were blocked with 5% skim milk for 30 min and incubated overnight at 4°C with primary antibodies diluted in 5% BSA [PARP (1:2000), cleaved-PARP (1:1000), cleaved caspase-3 (1:1000), cleaved caspase-7 (1:1000), cleaved caspase-8 (1:1000), Mcl-1 (1:1000), Bcl-2 (1:1000), Bak (1:1000), caspase-9 (1:1000), cleaved caspase-9 (1:1000), JAK2 (1: 1000), phospho-JAK2 (1:1000), STAT3 (1:2000), p-STAT3 (1:2000), cyclin D1 (1:2000), survivin (1:1000), vimentin (1:1000), AKT (1:2000), phospho-AKT (1:1000), CD49f (1:1000), CD44 (1:1000), ALDH1A1 (1:2000), Oct4 (1:1000), Nanog (1:1000), Sox2 (1:1000), P-gp (1:1000) and GAPDH (1:5000)], followed by incubation with HRP-conjugated anti-rabbit or mouse secondary antibodies (1:1000–1:10,000, Bio-Rad). Signal intensity was detected using a chemiluminescence kit (Thermo Fisher Scientific Inc., IL) on X-ray film (Agfa Healthcare, Belgium) and quantitated using AlphaEaseFC software (Alpha Innotech, CA).

***Immunoprecipitation assay***

To examine the impact of PITA on the interaction between Mcl-1 and phospho-ubiquitin, the Dynabeads™ Protein G Immunoprecipitation Kit (Thermo Fisher Scientific Inc., Rockford, IL) was utilized following the manufacturer's instructions. Cells were lysed in a lysis buffer (Pierce® IP) containing a cocktail of phosphatase and protease inhibitors. The supernatant was collected after centrifugation (14,000 g, 4℃, 10 min) and equal amounts (500 µg) were incubated with 2 µg of anti-Mcl-1 antibody conjugated to Dynabeads Protein G at 4℃ overnight. The protein complexes were recovered by boiling the beads in a mixture of SDS-PAGE sample buffer and elution buffer (1:1). Immunoblotting was conducted for Mcl-1 (1:1000) and phospho-ubiquitin (1:3000) using equal amounts of protein.

***Immunocytochemistry***

Cells on 8-well chamber slides (BD Biosciences) were fixed with 4% paraformaldehyde, washed with PBS, and permeabilized with 0.2% Triton X-100 for 13 min. The cells were then incubated overnight at 4°C with primary antibodies diluted in antibody-diluent (Dako, Denmark): Mcl-1 (1:100), ALDH1A1 (1:100), cytochrome C (1:100), phospho-histone H3 (1:100), P-gp (1:100), F-actin (1:100), Tom 20 (1:200), and ubiquitin (1:200). For the secondary antibody reactions, cells were stained with fluorescence-conjugated secondary antibodies [Alexa Fluor®-488 or -594 (Invitrogen, CA)] and then mounted with ProLong Gold Antifade Reagent with DAPI (Invitrogen). Images were acquired using Carl Zeiss LSM700 and LSM900 confocal microscopes (Weimar, Germany), and the fluorescence intensity was analyzed using the intensity profile tool ZEN black v3.0.

***In vitro mammosphere formation assay***

For the *in vitro* mammosphere-forming assay, BT549 (1×10^6^ cells/mL) and 4T1 (3×10^5^ cells/mL) cells were plated in ultralow attachment dishes (Corning, NY) and cultured in HuMEC basal serum-free medium (Gibco, MD), supplemented with B27 (1:50, Invitrogen), 20 ng/mL basic fibroblast growth factor (bFGF, Sigma), 20 ng/mL human or mouse epidermal growth factor (EGF, Sigma), 4 μg/mL heparin, 1% antibiotic-antimycotics, and 15 μg/mL gentamycin. The cultures were maintained at 37°C in an atmosphere of 5% CO_2_. The number and volume of mammospheres were determined using an Olympus CKX53 inverted microscope. Mammosphere volumes were calculated with the formula Volume=4/3*3.14(π)*r^3^ (r: radius).

***Limiting dilution assay***

4T1 cells were seeded into 24-well ultra-low attachment plates (Corning, NY) at densities of 1000, 600, 300, or 100 cells per well, with six replicates per condition. Cells were cultured in serum-free condition medium for mammosphere formation and treated with either vehicle control or PITA (1-5 μM). After 7 days of incubation at 37°C and 5% CO₂, wells were measured under a phase-contrast microscope and scored for the presence or absence of mammospheres. A well was considered positive if it contained at least one non-adherent spherical colony. The frequency of sphere-forming cells was calculated using the Extreme Limiting Dilution Analysis (ELDA) software (<http://bioinf.wehi.edu.au/software/elda/>) [1].

***Organoid culture and drug treatment***

Triple-negative breast cancer (TNBC) patient-derived cancer organoids (PDCOs) were obtained from the Korean Organoid Biobank and cultured according to the supplier’s protocol. For multiwell plate-based tumor organoid culture, organoids in suspension were centrifuged at 250×g for 5 min at 4°C to remove residual medium and cellular debris. The collected pellet was resuspended in cold, growth factor-reduced Matrigel (Corning) to maintain structural integrity and mimic the in vivo extracellular matrix environment. Matrigel–cell suspensions (20 µL) were then seeded into ultra-low-attachment 96-well plates (Corning, flat-bottom) and allowed to polymerize at 37°C for 30 min. After polymerization, 100 µL of Human Breast Cancer Organoid Complete Medium (MedChem Express, Cat. No. HY-K6101) was added to each well, and the plates were transferred to a humidified incubator at 37°C with 5% CO_2_. The culture medium was refreshed every 2–3 days by carefully aspirating half of the spent medium and replacing it with fresh medium to sustain optimal growth conditions.

Following 3 days of culture, organoids were treated with PITA and paclitaxel at various concentrations in the same medium. Drug-containing medium (100 µL) was added to each well, replacing half of the existing medium to facilitate gradual adaptation to drug exposure, while control wells received an equivalent volume of vehicle (DMSO). Organoids were incubated with the drugs for 1–2 weeks under standard culture conditions. At the endpoint, cell viability was assessed using the CellTiter-Glo 3D assay (Promega, WI), which quantifies ATP levels as a marker of metabolic activity. Luminescence was measured with a Varioskan LUX multimode microplate reader (Thermo Fisher Scientific, IL). For synergy assessment, organoids were treated with increasing concentrations of PITA (0-20 µM) and paclitaxel (0-20 nM) either alone or in combination for 7 days. The combination index (CI) and fraction affected (FA) were calculated using CompuSyn software (ComboSyn Inc., USA) based on the chou-Talalay method [2]. In this analysis, CI values < 1 indicate synergism, CI = 1 denotes an addictive effect, and CI > 1 indicates antagonism.

***Cell sorting and cytological centrifugation***

4T1 mammospheres were incubated with ACCUTASE^TM^ (Stemcell Technologies) for 15 min at 37°C. The dissociated cells were filtered through a 40 μm cell strainer (BD Biosciences) and incubated for 45 min at 37°C in Aldefluor assay buffer. Aldefluor-positive (ALDH1+) or -negative (ALDH1-) populations were sorted by FACS-Melody cell sorters (BD Bioscience). For immunocytochemistry, the sorted cells (2×10^4^) were attached to a glass slide by cytospin centrifugation (Hanil Science; Daejeon, Korea), fixed with 4% paraformaldehyde, washed with PBS, and permeabilized with 0.02% Triton X-100 for 13 min. The cells were then incubated overnight at 4°C with the primary antibodies [ALDH1A1 (1:50), Mcl-1 (1:100) or Tom 20 (1:200)] in antibody diluent. Following primary incubation, cells were stained with Alexa Fluor® 488- or 594-conjugated secondary antibodies at RT for 2 h and mounted using ProLong Gold Antifade Reagent with DAPI. Immunofluorescence mages were acquired using confocal microscopy.

***Detection of Reactive Oxygen Species (ROS) generation***

Reactive oxygen species (ROS) generation was monitored by staining cells with 2’, 7’-Dichlorodihydrofluorescin diacetate (DCFH-DA; Cell Biolabs Inc, CA). After treatment with PITA (1-5 μM) for 3 h, cells were further incubated with 1 mM DCFH-DA at 37°C for 30 min. The fluorescence of 2’,7’-Dichlorodihydrofluorescein (DCF), which is oxidized by intracellular ROS, was measured using flow cytometry at an excitation wavelength of 480 nm and emission of 530 nm. Data were analyzed using FlowJo software. For the measurement of mitochondrial ROS production, cells were stained with 5 μM dihydroethidium (DHE, Sigma-Aldrich) and analyzed by confocal microscopy.

***Measurement of mitochondrial membrane potential (∆Ψ m)***

Cells were treated with PITA (0-5 μM) for 48 h, followed by staining with 5 μM of the lipophilic dye 5,5',6,6'-tetrachloro-1,1',3,3'-tetraethyl-benzimidazolyl carbocyanine iodide (JC-1, Sigma-Aldrich) for 30 min at 37 °C. Fluorescence intensities were measured by flow cytometry: green fluorescence (indicative of loss of membrane potential, JC-1 monomers) at 535 nm, and red fluorescence (indicative of normal membrane potential, JC-1 aggregates) at 595 nm. A decrease in the red-to-green fluorescence intensity ratio was indicative of mitochondrial depolarization.

***Intracellular adenosine triphosphate (ATP) assay***

For intracellular ATP content analysis, cells were seeded in 96-well white/clear flat bottom plates (Corning). After 24 h of incubation, the cells were treated with PITA at 1 and 5 μM for 48 h. Following this, the cells were incubated with CellTiter-Glo® 2.0 ATP assay luminescent reagent (Promega) at 37°C for 10 min, according to the manufacturer’s instructions. ATP content was determined using a Varioskan LUX™ microplate reader (Thermo Fisher Scientific).

***Molecular Modeling and Docking Analysis***

Molecular docking studies were conducted using open platforms for protein-ligand virtual screening: GalaxySagittarius (https://galaxy.seoklab.org/), DockThor (https://www.dockthor.lncc.br/), and CB-Dock2 (https://cadd.labshare.cn/cb-dock2/). Upon completion of the docking simulation, the visualization and analysis of both 2D and 3D protein-ligand complexes, along with their predicted binding sites, were performed using UCSF chimera (https://www.cgl.ucsf.edu/chimera/) and BIOVIA Discovery Studio 2021 (<https://discover.3ds.com/discovery-studio-visualizer-download/>).

***Surface Plasmon Resonance (SPR) analysis***

SPR analyses were conducted at 25 ºC using iMSPR-ProX biosensors (icluebio) with HC1000M sensor chips (XanTec bioanalytics GmbH). The activated HC1000M chip was immobilized with recombinant human MCL1 protein (28 µg/mL) in 5 mM acetate buffer (pH 4.0) by amine coupling. The analyte was initially dissolved in 100% DMSO and then diluted with running buffer (10 mM phosphate, 140 mM NaCl, 2.7 mM KCl, 1 % DMSO, 0.005% Tween 20 under pH 7.4) to achieve concentrations of 25, 12.5, 6.25, 3.13, 1.56, 0.78, 0.39, and 0 µM. PITA and gossypol-acetic acid were then injected over the Mcl-1-immobilized chip at a flow rate of 50 µL/min. Each response curve was generated by subtracting the background signal from the control flow cell, and the binding data were analyzed using Tracedrawer Software.

***Allograft in vivo experiments and bioluminescence imaging (BLI)***

All animal procedures were carried out in accordance with animal care guidelines approved by the Korea University Institutional Animal Care and Use Committee (IACUC, KOREA-2021-0070). Five-week-old female BALB/c mice were obtained from the NARA Biotech Animal Center (Seoul, Korea), housed in a pathogen-free environment, and acclimated for 1 week prior to the study, with free access to food and water. Cells (1×10^5^) dissociated from 4T1 mammospheres were implanted subcutaneously into the fourth mammary fat pad of 6-week-old BALB/c female mice. When average tumor volumes reached 50 mm^3^, the animals were randomized into 2 groups (n=5/each group), and control vehicle (DMSO/corn oil, 1:9) or PITA (5 mg/kg·BW/day) was administered intraperitoneally every other day for 31 days. Tumor volumes were measured twice a week after the initial treatment using a caliper and calculated using the following formula; V=(Length×Width^2^)/2. After a period of 24 h following the final administration of PITA, the animals were anesthetized and subjected to NightOWL LB983 bioluminescence imaging (BLI) (Berthold Technologies, TN). D-luciferin sodium salt (BioVision Inc. CA) at a dose of 150 mg/kg·BW in 100 µL PBS was administered intraperitoneally as a substrate prior to BLI. Images were quantified (photons/sec) using the IndiGo™ software package. For lung metastasis analysis, lungs were harvested, fixed in 4% paraformaldehyde, embedded in paraffin blocks, sectioned at 5-μm thickness, mounted on positively charged glass slides and stained with hematoxylin and eosin (H&E). Images were acquired using a Zeiss Axio Scan.Z1 slide scanner and analyzed for metastatic lesions using ZEN software. For the syngeneic mouse metastasis model, 1×10^5^ cells dissociated from PacR-4T1 mammospheres were injected into the tail vein of BALB/c mice, followed by a single dose of intravenous control solvent, paclitaxel (4 mg/kg) or PITA (5 mg/kg).

***Serum biochemistry profiles for biomarkers of liver and renal injury***

At the time of sacrifice, blood samples were collected from each animal, and serum samples were obtained by centrifugation at 3000 rpm for 20 min. Serum enzyme activities for aspartate aminotransferase (AST), alanine aminotransferase (ALT), total bilirubin (TBL), blood urea nitrogen (BUN), and creatinine were evaluated by a serum biochemistry profiling service provided by DKKorea Inc. (Seoul, South Korea).

***Immunohistochemistry and in-situ localization of apoptosis (TUNEL)***

After removal, tumors were fixed in 4% paraformaldehyde and embedded in paraffin. Tissue sections of 5 µm thickness were mounted on positively-charged glass slides, deparaffinized with xylene, and dehydrated through a graded alcohol series to water. For antigen retrieval, tumor tissue sections were boiled in citric acid buffer (pH 6.0). The sections with primary antibodies [Ki-67 (1:100), cleaved caspase-3 (1:100), Mcl-1 (1:50), CD31 (1:100), phospho-STAT3 (Tyr705, 1:100), CD49f (1:100), CD44 (1:200), vimentin (1:200) or ALDH1A1 (1:100)] in antibody-diluent were incubated overnight at 4°C and then reacted with Alexa Fluor® 488- or 594-conjugated secondary antibodies at RT for 2 h, and mounted with ProLong Gold Antifade Reagent with DAPI. *In situ* TUNEL assays were conducted on tissue sections using a TUNEL kit (Roche Applied Sciences, GER) following the manufacturer’s instructions. All images were taken with a confocal microscope. The fluorescence intensities of images were analyzed using a histogram tool in the Carl Zeiss software package.

***MMP-2, MMP-9 and VEGF ELISA assay***

Matrix metalloproteinase-2 (MMP-2), MMP-9, and vascular endothelial growth factor (VEGF) levels in mouse serum were measured using ELISA kits (R&D Systems, MN) according to the manufacturer’s instructions. The quantities of MMP-2, MMP-9, and VEGF were determined by measuring the absorbance at 450 nm using a microplate reader.

***Wound healing and migration assay***

For kinetic migration analysis, cells were seeded to 80~90% confluency in 96-well plates (Essen ImageLock, Essen Biosciences, MI). Wound areas were created using a 96-pin Wound Maker device and then washed with culture media to prevent the reattachment of dislodged cells. After wound scratching, cells were treated with PITA, and images of the scratched fields were automatically acquired and recorded every hour for 30 or 48 h using an IncuCyte™ ZOOM® Kinetic Imaging System (Essen Biosciences). Relative wound density was analyzed using the IncuCyte™ Scratch Wound Cell Migration Software Module. A transwell assay was conducted to evaluate cell migration. Briefly, 3 × 10^5^ cells were seeded in the upper chamber (8-µm pore size; Corning Life Sciences) with serum-free medium, while the lower chamber contained 500 µl of FBS-supplemented medium. After 24 h, migrated cells were fixed in 4% formaldehyde, treated with methanol, stained with 0.1% crystal violet (Sigma-Aldrich), and counted under a microscope.

***Public dataset source and bioinformatics analysis***

The GENT2 dataset (GENT2: an updated gene expression database for normal and tumor tissues) (http://gent2.appex.kr/gent2/) was used to assess differential expression and prognostic significance based on tumor subtype. From this dataset, we downloaded a cohort of breast cancer patients and analyzed mRNA expression and patient survival according to the molecular subtypes of ER, PR, and HER2 status. Overall survival regression data were acquired by Kaplan-Meier analysis using GraphPad Prism 9.0 software, after categorizing patients into high- and low-expression groups in the GENT2 dataset and TCGA (The Cancer Genome Atlas) cohorts obtained from UCSC Xena (<http://xena.ucsc.edu>). RNA-Seq data from the publicly available Gene Expression Omnibus (GEO, https://www.ncbi.nlm.nih.gov/geo/) dataset GSE162187 [3], which contains transcriptomic profiles from breast cancer patients classified as sensitive or resistant to neoadjuvant chemotherapy, include 13 taxane-resistant and 9 taxane-sensitive samples. For our analysis, raw expression data were normalized to transcripts per million (TPM) and log2-transformed for downstream statistical evaluation. The expression levels of MCL1 isoforms ENST00000369026 and ENST00000620947 were compared between the two response groups, and statistical significance was assessed using an unpaired two-tailed t-test. Kaplan-Meier survival curves were calculated up to 200 months, with *p*-values obtained through the *log-rank* test. Correlations of mRNA gene expression levels were analyzed using Pearson's correlation coefficient (R).

***Statistical analysis***

All data were analyzed using GraphPad Prism 9.0 statistical software (San Diego, CA). The results are presented as mean ± SEM of at least three independent experiments. Depending on the experimental design, comparisons were conducted using unpaired Student’s t-tests, one-way or two-way ANOVA, and log-rank tests for survival analyses, with Bonferroni’s post hoc test applied for multiple group comparisons. Statistical significance was defined as p < 0.05. In addition to p-values, the practical significance of our findings was evaluated by calculating effect sizes (e.g., Cohen’s d, Hedges’ g, and Eta-squared [η^2^]) and by presenting 95% confidence intervals, particularly for key comparisons in gene expression, cell viability assays, and survival outcomes.

**References**

1. Hu Y, Smyth GK. ELDA: extreme limiting dilution analysis for comparing depleted and enriched populations in stem cell and other assays. J Immunol Methods. 2009;347:70-8.

2. Chou TC, Talalay P. Quantitative analysis of dose-effect relationships: the combined effects of multiple drugs or enzyme inhibitors. Adv Enzyme Regul. 1984;22:27-55.

3. Barron-Gallardo CA, Garcia-Chagollan M, Moran-Mendoza AJ, Delgadillo-Cristerna R, Martinez-Silva MG, Villasenor-Garcia MM, et al. A gene expression signature in HER2+ breast cancer patients related to neoadjuvant chemotherapy resistance, overall survival, and disease-free survival. Front Genet. 2022;13:991706.
